# Background-free quantitative phase imaging with adaptive-optics surface plasmon resonance holographic microscopy

**DOI:** 10.1038/s41377-026-02362-x

**Published:** 2026-07-14

**Authors:** Siqing Dai, Mengmeng Zhang, Yushan Shen, Haoyu Xu, Li Ren, Hua Lu, Jiwei Zhang, Gerd Ulrich Nienhaus, Jianlin Zhao

**Affiliations:** 1 Key Laboratory of Light Field Manipulation and Information Acquisition, Ministry of Industry and Information Technology, and Shaanxi Key Laboratory of Optical Information Technology, School of Physical Science and Technology, Northwestern Polytechnical University, Xi’an, China; 2 Key Laboratory for Space Bioscience and Biotechnology, School of Life Science, Northwestern Polytechnical University, Xi’an, China; 3 Institute of Applied Physics, Karlsruhe Institute of Technology, Karlsruhe, Germany; 4 Institute of Biological and Chemical Systems and Institute of Nanotechnology, Karlsruhe Institute of Technology, Eggenstein-Leopoldshafen, Germany; 5 Department of Physics, University of Illinois at Urbana-Champaign, Urbana, IL USA

**Keywords:** Phase-contrast microscopy, Imaging and sensing

## Abstract

Quantitative phase imaging (QPI) in the near field is a powerful tool for visualizing nanoscale structures in low-dimensional materials, dielectric mixtures and biological cells. Although near-field QPI offers extremely high sensitivity, phase aberrations of the optical system can pose serious limitations. Overcoming these problems, we introduce an adaptive optics approach that takes advantage of the complex amplitude measured by digital holographic microscopy (DHM). By using a spatial light modulator as a beam shaping device, our method allows for in-situ, accurate, fast and flexible aberration correction by quantifying wavefront distortions in terms of Zernike modes, and pre-compensating them with a spatial light modulator. For validation, we demonstrate near-field phase imaging with adaptive-optics surface plasmon resonance holographic microscopy (AO-SPRHM) on microstructured test samples and live cells. With a total correction time below 1 s, background-free time-lapse imaging over many hours becomes feasible. The approach can be easily transferred to other phase imaging techniques, including transmission, reflection and total internal reflection DHM as well as related modalities.

## Introduction

Recent years have witnessed the rapid development of quantitative phase imaging (QPI), and its broad application in diverse fields such as materials science, complex fluid visualization, biological imaging, and medical diagnosis^1,2^. Several approaches have been devised for QPI, including digital holographic microscopy (DHM)^3^, transport-of-intensity equation (TIE)-based QPI^4^ and Fourier ptychographic microscopy (FPM)^5^, and so on. DHM, which is based on measuring interference fringes of object and reference waves to numerically reconstruct phase-contrast images, is a well-established and popular technology due to its noninvasiveness, wide-field and real-time measurement capabilities^3,6,7^. Surface plasmon resonance holographic microscopy (SPRHM) is an exciting DHM variant that enables highly sensitive near-field imaging^8^. It integrates a surface sensing technique, surface plasmon resonance (SPR), with DHM, so that SPR intensity- and phase-contrast images of samples located close to an SPR-active metal surface can be simultaneously acquired. SPR features an extremely high refractive index (RI) detection sensitivity (up to 10^-8^ RIU) within the evanescent field of about 100–200 nm thickness^8^. Accordingly, SPRHM has found wide application in measurements of droplet evaporation^9^, 2D materials characterization^10^, live-cell imaging^11–13^, and nanoparticle analysis^14^.

Optical aberrations, which are inevitably present in any real imaging device including SPRHM, can greatly degrade the quality of near-field QPI and, consequently, the accuracy of the measurement. The off-axis interferometric configuration between object and reference waves inherently introduces a significant tilt aberration, and other modes of aberration, such as astigmatism, coma, trefoil and spherical aberration may also exist in optical systems due to imperfect optical components, system misalignment, and RI mismatch between the immersion fluid and the objective lens. Furthermore, time-varying wavefront aberrations, which may arise from mechanical instabilities, air turbulence, mechanical vibrations and sample variations, can further degrade the system performance in long-term measurements.

So far, various approaches have been pursued to correct optical aberrations in imaging systems. Hardware solutions for compensating the quadratic aberration in non-telecentric systems include insertion of a cubic beam splitter^15^ or a programmable lens^16,17^. A wide range of numerical solutions for the recovery of image information from distorted background have been devised^18–23^. Still, computational approaches face limited aberration mode correction, slow correction speed and the need for considerable computational resources. Previously, we introduced the double-exposure (DE) method to remove background phase aberrations in SPRHM^10,11,14^. With this differential technique, two digital holograms are measured in sequence, one with and another one without a sample, so that subtraction of the reconstructed phase images cancels background phase distortions^24,25^. Despite its effectiveness, the DE method has clear drawbacks. It relies on removing the sample from the imaging system without modification of system properties, which is not always feasible. Furthermore, at least two images have to be acquired in succession, extending the total measurement time and introducing errors due to time-varying aberrations, which is especially relevant for long-term experiments.

Thus, there is great demand for an accurate, fast and versatile technique that can compensate phase aberrations in SPRHM. In this context, the use of adaptive optics (AO) offers great opportunities to dynamically correct wavefront distortions. AO-based approaches involve two steps, (1) quantitative assessment of the aberrations, either by direct wavefront sensing or indirect (‘sensor-less’) optimization, and (2) employing correction elements such as deformable mirrors and spatial light modulators (SLMs) to compensate the identified aberrations^26^. The application of AO in optical (super-resolution) fluorescence microscopy has been impressively advanced to enhance the image quality^27–31^. AO implementations in QPI are still scarce. In a recent publication, AO was combined with dark-field imaging to expand the dynamic range of QPI^32^, and in another piece of work, AO correction was implemented in FPM, achieving annular illumination optimization and high resolution QPI^33^.

Here, we integrate AO with SPRHM (AO-SPRHM) to eliminate phase aberrations in near-field phase imaging. Leveraging the complex amplitude measurement in SPRHM, we employ modal wavefront sensing to decompose the wavefront distortions into Zernike modes. Their coefficients are determined by fit algorithms that aim at minimizing a suitable image metric. The calculated phase pattern is uploaded to an SLM to compensate the wavefront aberrations, resulting in direct SPR phase imaging. As demonstrated by live-cell applications, AO-SPRHM greatly facilitates background-free imaging of cellular adhesion dynamics. More importantly, our technique paves a new way to correct optical aberrations in the QPI field and various other imaging modalities in general.

## Results

### Principle of AO-SPRHM

**Fig. 1:**
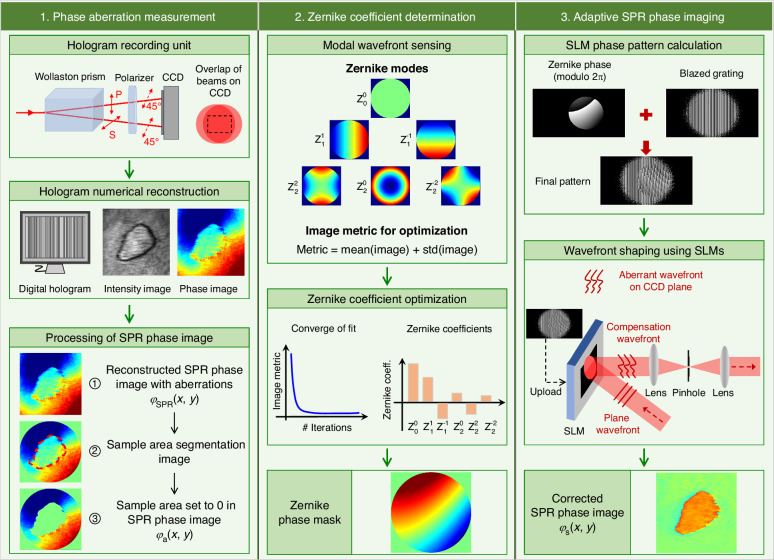
Principle of AO-SPRHM. Step 1: Light reflected by the SPR chip is split into *p*- and *s*-polarized components to generate a digital hologram. SPR intensity and phase-contrast images are numerically reconstructed from the recorded holograms. In the SPR phase image, the sample region is identified by segmentation, and phases are set to zero in this region. Step 2: Modal wavefront sensing is employed to determine Zernike coefficients that describe the optical aberrations. By using an iterative optimization algorithm, the Zernike mode coefficients are varied until the mean of absolute values and standard deviations of the phases outside the sample region are minimized. A Zernike phase mask is calculated that allows to compensate the optical aberrations. Step 3: The phase mask, superimposed with a blazed grating, is uploaded onto the SLM, so that the AO-SPRHM irradiates the sample with a modulated wavefront compensating optical aberrations, resulting in aberration-free SPR phase imaging

Our AO-SPRHM implementation involves three distinct steps, measurement of phase aberrations, determination of Zernike coefficients and AO-based phase correction, as depicted schematically in Fig. 1.

In the first step, we measure phase aberrations with our hologram recording unit (Fig. 1). In this device, *p*- and *s*-polarized light reflected from the SPR chip pass through a Wollaston prism, from which the two components exit under a mutual angle of ~2.1° at 632.8 nm. Since only *p*-polarized light can excite SPR, this component carries sample information and thus acts as the object beam, and the *s*-polarized component serves as the reference beam. After passing through a linear polarizer at 45°, both components have the same polarization and interfere in the overlap region; a CCD camera captures the digital hologram in the overlap region. The complex amplitude distribution of the object wave is computed with a numerical algorithm, from which the SPR intensity- and phase-contrast images can be reconstructed, as detailed in Supplementary Note 1. Here, we are only interested in the SPR phase images, which are severely impaired in the presence of optical aberrations. The measured SPR phase image, *φ*_SPR_(*x*, *y*), with pixel coordinates, *x*, *y*, along the horizontal and vertical dimensions, respectively, is a sum,

1 $${\varphi }_{{\rm{SPR}}}(x,y)={\varphi }_{{\rm{s}}}(x,y)+{\varphi }_{{\rm{a}}}(x,y)$$

of the correct, aberration-free SPR phase image of the sample, *φ*_s_(*x*, *y*), and a background image carrying the phases due to aberrations, *φ*_a_(*x*, *y*). To extract *φ*_a_(*x*, *y*) from *φ*_SPR_(*x*, *y*), the sample region in *φ*_SPR_(*x*, *y*) is segmented, and all phases in this region are set to zero to generate a phase image that is taken for wavefront sensing. In this way, we effectively protect the sample information from potential artifacts introduced by the ensuing wavefront optimization step. Notably, even with samples featuring dense cell layers and only small background regions, we found considerable robustness in the aberration correction by AO-SPRHM (Supplementary Fig. S1).

In the second step, modal wavefront sensing is performed, with *φ*_a_(*x*, *y*) as input, by decomposing the aberrant phases in terms of Zernike modes,

2 $${\varphi }_{{\rm{a}}}(x,y)=\mathop{\sum }\limits_{n,m}{C}_{n}^{m}{Z}_{n}^{m}(\rho ,\theta )$$

where ${Z}_{n}^{m}$ and ${C}_{n}^{m}$ denote the Zernike modes and their corresponding coefficients, respectively. The polar coordinates, radius, *ρ*, and polar angle, *θ*, of the Zernike polynomials are transformed into the corresponding Cartesian pixel coordinates, *x*, *y*. Based on prior examination of the Zernike modes required for wavefront correction (Supplementary Fig. S2), we selected a combination of the first six Zernike modes, which faithfully describe the phase aberrations of AO-SPRHM system. The optimal Zernike coefficients are determined by heuristic optimization algorithms, which are efficient problem-solving tools offering high speed and scalability and providing near-optimal solutions^34^. We have implemented five different classical fitting algorithms in this work (see Materials and methods), hill climbing (HC), stochastic parallel gradient descent (SPGD), simulated annealing (SA), particle swarm optimization (PSO) and the genetic algorithm (GA). Within these algorithms, the core procedure is to generate a Zernike phase mask, *φ*_m_(*x*, *y*), and add it to the distorted background phase image, *φ*_a_(*x*, *y*), to generate an iterative phase image, *φ*_i_(*x*, *y*),

3 $${\varphi }_{{\rm{i}}}(x,y)={\varphi }_{{\rm{m}}}(x,y)+{\varphi }_{{\rm{a}}}(x,y)$$

This image is quantitatively evaluated by an image metric *IM*,

4 $$IM=M+S$$

with mean of absolute values,

5 $$M=\frac{1}{XY}\mathop{\sum }\limits_{1}^{X}\mathop{\sum }\limits_{1}^{Y}|{\varphi }_{i}(x,y)|$$

and standard deviation,

6 $$S=\sqrt{\frac{1}{XY-1}\mathop{\sum }\limits_{1}^{X}\mathop{\sum }\limits_{1}^{Y}{[{\varphi }_{i}(x,y)-\overline{{\varphi }_{i}}]}^{2}}$$

*X* and *Y* are the total pixel numbers along the horizontal and vertical dimensions, respectively. Despite its simplicity, this image metric is most suitable as the loss function in the iterative optimization. It converges to zero upon successful compensation of the aberrated background phase, and the best-fit Zernike coefficients are subsequently used to generate the phase mask for image correction. Notably, to accelerate the fitting procedure, the SPR images (960 × 960 pixels) are compressed by a factor of five, which is sensible since our low-index Zernike polynomials introduce only low-frequency variations. Additionally, our SPR phase image processing approach is only valid for a loss function (*IM*) that converges to 0 in the fit. If that is not the case, it will be easy to include only background pixels, i.e., those outside the masked region of the sample, in the optimization.

In the third step, the Zernike phase mask, superimposed with a blazed grating, is uploaded onto the SLM. A 4 *f* system with a pinhole in-between allows the zeroth diffraction order of the *s*-polarized component and the first diffraction order of the *p*-polarized component of the light reflected/diffracted by the SLM to pass through and filters out higher diffraction orders. As a result of pre-compensation of wavefront distortions by the SLM, a *p*-polarized plane wavefront effectively illuminates the sample on the SPR chip, while the *s*-polarized component remains unmodified. Then, an aberration-free SPR phase image of the sample can be acquired.

Detailed descriptions of the AO-SPRHM setup and the optimization algorithms are provided in Materials and Methods and the supplement.

### Validation of AO-SPRHM with a test sample

**Fig. 2:**
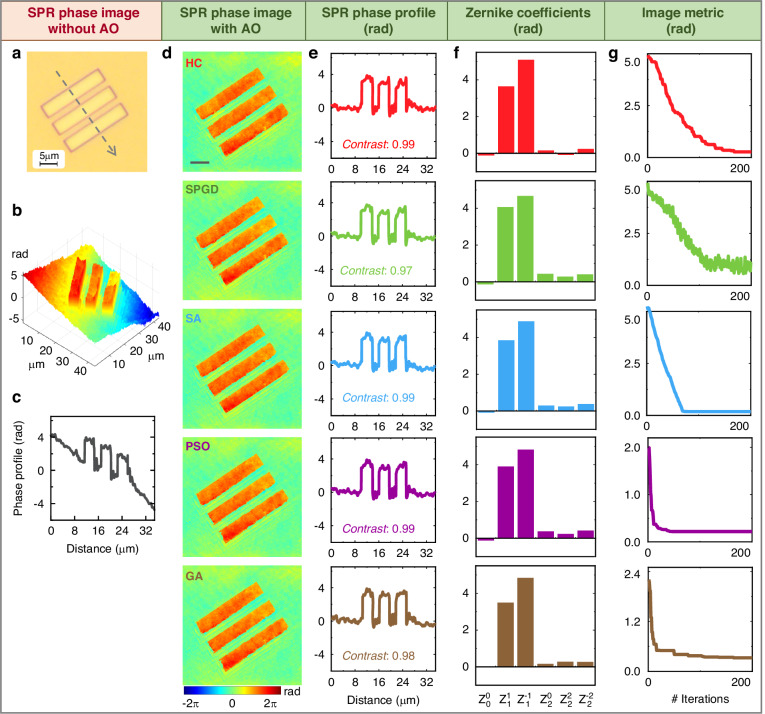
Near-field QPI on an EBL-structured photoresist layer on gold using AO-SPRHM. **a** Optical image of the test sample. **b** Pseudo-3D depiction of the SPR phase image and **c** phase profile along the dashed arrow in **a** before aberration correction. **d** Aberration-free SPR phase images extracted with five different optimization algorithms, scale bar, 5 μm. **e** Corresponding phase profiles and their contrast, (|${\bar{\varphi }}_{{\rm{s}}}$| – | ${\bar{\varphi }}_{{\rm{b}}}$ | )/(|${\bar{\varphi }}_{{\rm{s}}}$ | + |${\bar{\varphi }}_{{\rm{b}}}$ | ), where ${\bar{\varphi }}_{{\rm{b}}}$ is the corrected background phase and the overbar denotes pixel averaging. **f** Best-fit Zernike coefficients, and **g** *IM* minimization as a function of the number of iterations

To demonstrate the effectiveness of our method, we first examined our AO-SPRHM by imaging a test structure. For this sample, a ~100-nm thick (negative) photoresist layer on top of a 45-nm gold layer was structured by electron beam lithography (EBL). After etching, three rectangular photoresist bars of 4.0 × 20.0 µm^2^ remained, as shown by the optical image in Fig. 2a. The SPR excitation angle was adjusted to 36.3°, as appropriate for air as the top layer^35^. Upon plasmon excitation in the gold layer, the high-RI photoresist coating gives rise to a strong phase shift with respect to outside regions and, thus, a high-contrast SPR phase image. We first captured a hologram and reconstructed a SPR phase image without any aberration correction, showing that the sample phase pattern is superimposed on a strong background variation due to optical aberrations (Fig. 2b, c). Next, we applied the adaptive correction using our five optimization algorithms; the resulting SPR phase images are shown in Fig. 2d. Compared with the reference, all five algorithms yield greatly improved phase images; the sample structure is reproduced with good contrast against a flat background. As anticipated from the successful correction of the SPR images (Fig. 2d), the best-fit Zernike coefficients (Fig. 2f) are similar for the different fit algorithms (Supplementary Table S1). Specifically, the ${Z}_{1}^{1}$ (tip) and ${Z}_{1}^{-1}$ (tilt) modes have markedly larger coefficients than the other Zernike modes and thus play a key role, as expected from looking at Fig. 2b. The loss function, *IM*, drops with the number of iterations and finally converges toward a plateau (Fig. 2g). Taken together, these results successfully validate the phase aberration correction capabilities of our AO-SPRHM system, so that it can readily be used for QPI-based sample characterization.

### Near-field QPI of live human breast cancer cells

**Fig. 3:**
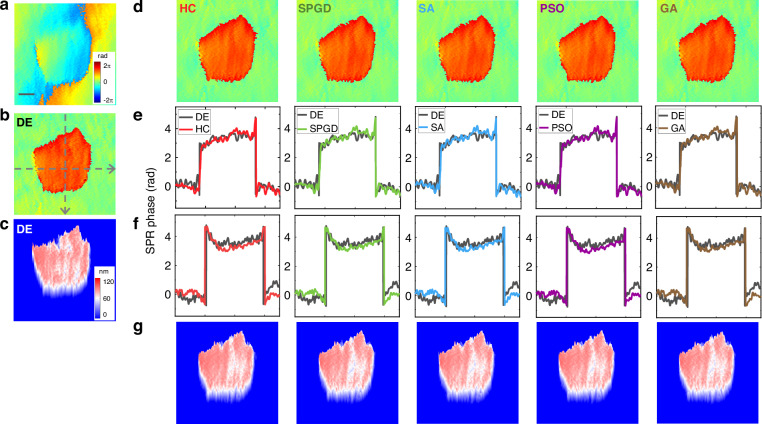
Near-field QPI of a live breast cancer cell sample. **a** SPR phase image without aberration correction. **b** Aberration-corrected SPR phase image using the DE method. **c** Cell adhesion gap image calculated from the phase image in panel **b**. **d** AO-SPRHM-generated phase images. Phase profiles along the **e** horizontal and **f** vertical arrows shown in **b**. **g** Cell adhesion gap images calculated from the phase images in **d**. Scale bar (applies to all cell images), 5 μm

As a surface-sensitive optical imaging technique, SPRHM is particularly advantageous for monitoring cell adhesion via the basal cell membrane, providing valuable information about cell differentiation, spreading, and apoptosis^36^. Therefore, we next applied AO-SPRHM to live imaging of adherent cells of the human breast cancer cell line MDA-MB-231. We adjusted the incident angle for SPR excitation to 55.4°, as appropriate for samples embedded in aqueous solution (cell culture medium). Figure 3a presents an SPR phase image of a cell sample using regular SPRHM, showing the phase signature of the cell riding on a strong background phase distribution. To compare AO-SPRHM with the DE method^11^, we next measured cell images using our five correction algorithms, and at the end, we moved the cell out of the field of view to collect a background image for DE. Comparison of the raw image (Fig. 3a) and the DE-corrected image (Fig. 3b) reveals effective removal of the background phases. The retained phase variations of the cell result from the varying thickness of the adhesion gap between the basal cell membrane and the gold substrate. An adherent cell is known to bind to the gold substrate via focal adhesions, multi-protein complexes that locally link the intracellular cytoskeleton to the cell exterior, here the gold surface. Focal adhesions are very dynamic; they continuously disassemble and form anew as a cell migrates across a surface^37,38^. These processes have been visualized earlier by super-resolution fluorescence imaging^39^, and QPI is a powerful approach to extend such measurements to significantly faster timescales. While there is closest cell-surface contact at the site of a focal adhesion, the adhesion cleft opens between these points of attachment, with a mean gap width of 50 – 100 nm^40^. To extract the gap width from QPI data, the measured phases have to be converted into the vertical gap width. To this end, we use a multilayer SPR model and the Fresnel equations to calculate the reflection coefficient as a function of the gap thickness, from which the mapping between the phase and the gap width can be obtained (Supplementary Note 2). The resulting pseudo 3D image of the adhesion cleft reveals large variations in the gap width (Fig. 3c).

In Fig. 3d–g, we present QPI results of the same cell, as obtained by AO-SPRHM using our five aberration correction algorithms. The SPR phase images (Fig. 3d) are all similar to those obtained by the DE method, and quantitative comparison of the mean and standard deviation of the background after correction yields similar results (Supplementary Table S2). Likewise, the phase profiles along the horizontal and vertical directions, plotted in Fig. 3e, f, respectively, agree overall very well with those of DE. However, there are abrupt changes of the SPR phase visible at the cell edges. This artifactual behavior occurs for two reasons: (1) Our SPR model is based on a layered structure (Supplementary Fig. S3a), which obviously cannot faithfully describe regions near the cell edge. (2) The incident angle of the light wave was adjusted for minimal reflection and thus effective surface plasmon wave (SPW) excitation at the cell edges, in line with previous work^11^. The transverse propagation of SPWs distorts the SPR phase images and causes phase errors, thus deteriorating the SPR image quality and spatial resolution^14^. By limiting the adverse effect of pronounced SPW excitation to the cell edges, we ensure that we capture precise phase images of the cells except in the boundary regions. The corresponding maps of the adhesion gap are depicted in Fig. 3g. When measuring more cell samples, we found out that AO-SPRHM demonstrates more robustness compared to DE in some cases (Supplementary Fig. S4). Taken together, our results indicate that our AO-SPRHM implementation performs very well in live-cell QPI. Furthermore, comparison of various optimization algorithms shows that the hill climbing algorithm outperforms other fitting methods in terms of correction speed and accuracy (Supplementary Fig. S5).

### Long-term near-field QPI of a live osteoblast cell

**Fig. 4:**
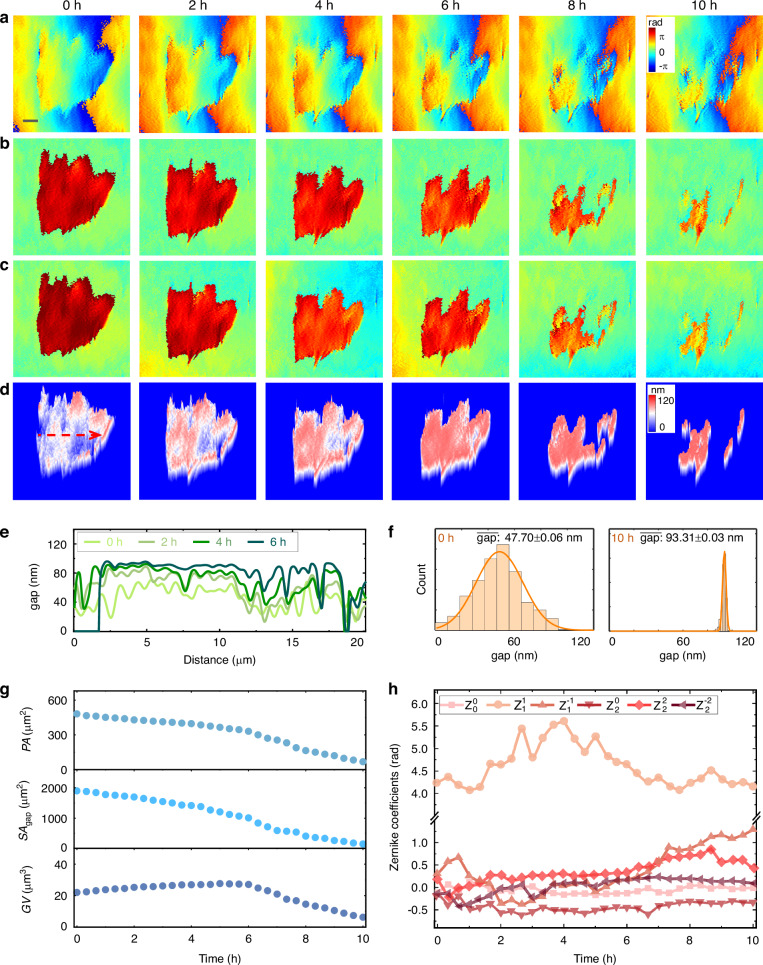
Long-term near-field phase imaging of a live osteoblast cell. **a** Raw SPR phase images measured by regular SPRHM. Aberration-free phase images measured by **b** AO-SPRHM and **c** DE. **d** Cell adhesion gap images demodulated from **b**. **e** Profiles of the cell adhesion gap along the dashed arrow in **d** at 0, 2, 4 and 6 h. **f** Histograms and Gaussian fits of cell adhesion gap at 0 and 10 h. Temporal variations of **g** *PA*, *SA*_gap_ and *GV* and **h** Zernike coefficients measured every 20 min. Scale bar (applies to all images): 5 μm

An important application of AO-SPRHM is long-term monitoring of cellular activity, which requires continuous compensation of time-varying aberrations. As an example, we have visualized a cell from the mouse osteoblast precursor cell line MC3T3-E1, using AO-SPRHM without and with aberration correction (using the HC algorithm for best performance). Before the cell sample was moved into the field of view, an initial reference hologram was recorded. Afterward, the cell was gently moved into the field of view and imaged using regular SPRHM and AO-SPRHM, with phase correction every 20 min for a total period of 10 h. The sequence of uncorrected phase images (Fig. 4a) reveals marked changes in the morphology of the cell-surface interface, indicating that the cell detaches from the substrate over time. The strong background phase distribution, however, precludes any precise QPI or even phase mapping of the gap structure. Close inspection shows phase variations over the course of the experiment due to environment fluctuations and optical system instabilities, calling for continuous aberration correction.

The corresponding phase images acquired by AO-SPRHM are shown in Fig. 4b. The gradual detachment of the cell from the substrate due to weakened cell-substrate interactions is clearly visible in the aberration-corrected phase images. By contrast, the image quality of the corrected SPR phase images acquired by DE (Fig. 4c) gradually degrades over time due to background drift, causing background phase variations and, consequently, phase errors in the cellular region. The comparison of AO-SPRHM and DE demonstrates the inherent advantage of AO-SPRHM, offering in-situ correction and thus immunity to dynamically varying aberrations between exposures. AO-SPRHM with continuous aberration correction permits quantitative analysis of cell adhesion kinetics, which is not feasible with the DE method due to background instabilities. Specifically, the cell adhesion gap can easily be reconstructed from the phase maps using AO-SPRHM (Fig. 4d), and the images and the profiles (Fig. 4e) clearly reveal a widening distance between the osteoblast cell and the gold substrate. In the end, the local SPR signal vanishes as the cell-surface separation becomes larger than the penetration depth of the evanescent field (~100 nm). From histograms of the pixel counts versus the gap width, we find an average gap width increase from 47.70 ± 0.06 nm at the beginning to 93.31 ± 0.03 nm after 10 h (Fig. 4f). Furthermore, we have analyzed the time course of three physical parameters introduced earlier to describe the adhesion cleft (Fig. 4g)^41^: (i) the projected area, *PA*, i.e., the area of cell projected onto the substrate plane, (ii) the surface area, *SA*_gap_, i.e., the sum of the upper and lower surfaces of the cell gap, and (iii) and the gap volume, *GV*. The first two parameters follow a continuously descending trend as the cell gradually loses contact to the surface, whereas *GV* increases up to 6 h, showing that the widening of the gap is overcompensated by the loss in adhesion area in this period. Finally, the volume decreases as the gap widens beyond the range of the evanescent field, causing an effective decay of the volume accessible to the experiment. Figure 4h shows the dynamic variations of the coefficients of the six selected Zernike modes over time. Note that, in contrast to Figs. 3 and 4, where *x*-tilt (tip, ${Z}_{1}^{1}$) and *y*-tilt (tilt, ${Z}_{1}^{-1}$) play a major role, *x*-tilt is predominant here, as is obvious from the background phase pattern in Fig. 4a. Especially, the tilt modes are found to vary significantly over time, demanding continuous updates of the phase correction. Background phase drifting was investigated based on the data in Fig. 4h. By analyzing frequency spectra computed by Fast Fourier Transform (FFT) (Supplementary Fig. S6), the main drift component was found to have a timescale of ~3700 s, indicating that the chosen correction interval of 1200 s is fast enough to capture the drifts. We performed another continuous aberration correction experiment over 2 h, with a significantly shorter time interval for correction, i.e., 300 s. We observed phase fluctuations with timescales of 2500–3800 s (Supplementary Fig. S7), demonstrating that phase drifting of our system is slower than achievable correction intervals.

## Discussion

Here, we have presented an AO-SPRHM implementation for background-free QPI. The unique advantage of our DHM-based approach is that no additional expensive hardware such as wavefront sensors or complex calibrations is required. We measure the aberrated wavefront in situ in empty regions of the sample, which allows for continuous correction of time-varying aberrations in time-lapse experiments over many hours (Fig. 4). Therefore, SLM-based AO-SPRHM enables in in-situ, flexible and real-time aberration correction, which is essential for imaging experiments with dynamic monitoring. Moreover, the hologram recording unit which consists of Wollaston prism, polarizer and CCD in the optical system ensures common-path interference between object and reference waves, enhancing the compactness of the optical setup and offering immunity to environmental instabilities. In addition to the hardware-based DE method, we compared AO-SPRHM with a purely computational method, i.e., sparse optimization (SO)^20^. Comparison of the corrected SPR phase images of cell samples shows that AO-SPRHM outperforms the SO method in terms of both correction accuracy and speed (Supplementary Fig. S8). AO-SPRHM is reliable and robust, and works even with samples consisting of dense cell layers and smaller empty islands (Supplementary Fig. S1). For completely automatic background correction, these regions can easily be identified by automatic segmentation. To underscore the general applicability of our method to diverse samples, we have also investigated samples of low-dimensional graphene material and different cultured cells (K562 lymphoblasts, human leukemia cell line) by using AO-SPRHM (Supplementary Fig. S9).

However, AO-SPRHM still has limitations that we will address in the future. First, the method relies on the presence of a certain fraction of background area, from which background phase aberrations can be determined for adaptive correction. To investigate the minimum background ratio needed in the fitting process, we artificially varied the background ratio in cell images and studied the resulting correction performance (Supplementary Note 3), and found that the minimum fraction of background pixels (area) for stable aberration correction is ~0.35. A second, important issue concerns the proper Zernike mode selection for compensating phase aberrations. Modal wavefront sensing shows that the aberrations are well captured by the first six Zernike modes, i.e., piston, tip, tilt, defocus and *x-/y*-astigmatism. These are continuous, smoothly varying phase distributions that can be reliably extrapolated from background regions of the sample to the regions of interest. By adding the next five (more structured) Zernike modes (*x-/y*-coma, *x-/y*-trefoil and primary spherical), the aberration correction worsened, yielding non-uniform background and clear artifacts (Supplementary Fig. S2), regardless of the fitting method. It appears that the additional testing modes are not necessary for our optical system; they only disturb the optimization process and introduce errors. In general, one has to maintain flexibility and include all modes needed for aberration correction of the particular optical system at hand. For example, restricting Zernike functions to the first six modes will bias the sample phase if coma (${{\rm{Z}}}_{3}^{-1},\,{{\rm{Z}}}_{3}^{1}$) is present in the SPRHM system (Supplementary Note 4). To provide “plug-and-play” capability when applying the proposed technique to other imaging setups, fitting algorithms and software can be enhanced with adaptive selection of the required Zernike modes. Starting from low-order modes, higher-order testing modes can be sequentially included to examine if a better performance can be achieved. Weighting factors can be applied to the Zernike coefficients to adjust the sensitivities of higher-order Zernike modes. This process can be implemented in a completely automatized fashion.

Fitting algorithms play an important role in identifying optimal Zernike coefficients, and thus are pivotal determinants of the AO-SPRHM performance in terms of correction speed and accuracy. Comparing five classical fitting algorithms, we found that the simple HC algorithm performed best in terms of speed and fit quality, presumably because the simple metric based on mean and variance of the background phase image was employed as a loss function. Notably, HC is a local search algorithm that cannot cope with multiple maxima, so it requires a convex landscape in solution space. In this work, the HC method always found the precise correction of distorted SPR phase images, regardless of the initial values of the Zernike coefficients. This is expected, given that only the first six Zernike modes were employed in the fitting process. However, if higher-order Zernike modes are needed to describe aberrations in the optical system, multiple minima may appear in the landscape, and the performances of the different algorithms will have to be re-examined. Considering the sparse and smooth aberrant background in our SPRHM system, overfitting could become a problem. To this end, *L*_1_ and *L*_2_ regularization, which aim to mitigate the overfitting problem, were tested using the HC method (Supplementary Note 5). However, we found that the stopping rules implemented in our fitting algorithms effectively already prevent overfitting, given that the proper set of Zernike modes and sufficient background area are available.

To conclude, we have applied adaptive background phase correction to SPRHM and have demonstrated its effectiveness with proof-of-concept experiments on test samples and live cells. This method can readily be applied to various other QPI techniques. For example, dual-wavelength capability can be implemented into our AO-SPRHM by adding a second wavefront shaping unit at 690 nm to the system to enable dual-parameter characterizations^12,13^. Our SLM-based adaptive correction can also be employed in transmission DHM for live-cell imaging^42^. Moreover, in incoherent digital microscopy, our strategy can be embedded into the computational adaptive optics (CAO) approach in the image reconstruction process to eliminate aberrations and enhance the imaging quality^43^.

## Materials and methods

### AO-SPRHM system

A schematic of our AO-SPRHM system is shown in Supplementary Fig. S16. A He-Ne laser beam with a wavelength of 632.8 nm is collimated by a lens and adjusted to linear polarization at 45° by a half-wave plate. This beam is reflected by a phase-type SLM (LETO-3, HOLOEYE, Germany), which is programmed to modulate the wavefront of the *p*-polarized component. A blazed grating is always superimposed onto the desired phase pattern of the SLM to reflect the *p*-polarized component into the first diffraction order. A 4 *f* system, which comprises Lens1 (*f*_1_ = 100 mm), Lens2 (*f*_2_ = 150 mm) and a pinhole, passes the zeroth diffraction order of the *s*-polarized component and the first order of the *p*-polarized component, and excludes higher diffraction orders. Lens 3 focuses the beam into the back focal plane of a high-NA microscope objective (MO, APON100×HOTIRF, OLYMPUS, Japan), allowing wide-field illumination at the sample plane. The SPR-active chip (described below) is coupled to MO by RI-matching mounting medium (diiodomethane, Cargille Labs, USA). The light beam is incident on the SPR-active gold layer under the proper angle for surface plasmon excitation. The reflected beam, is collimated by Lens4 and enters the hologram recording unit, where the *s*- and *p*-polarized components pass a Wollaston prism (model 68-820, Edmund Optics, USA) so that they exit under a mutual angle of ~2.1°. After passing a polarizer at 45°, they interfere and form a hologram on the CCD camera chip. The optical path of the microscope consists of three successive 4 *f* systems that image the SLM plane to the CCD plane to capture aberration-corrected images (Supplementary Fig. S17).

From the digital hologram, a phase image is reconstructed. Pixels in regions without cells attached to the SPR chip carry phases that arise solely from aberrations. This information is used by our software algorithms to iteratively determine a phase map arising solely from optical aberrations. The (inverted) best-fit phase pattern is uploaded onto the SLM to compensate the aberrant wavefront of the object beam, so that an aberration-corrected SPR phase image can be reconstructed from the recorded hologram.

### Fitting algorithms

We briefly describe the five algorithms that were implemented to identify the optimal Zernike coefficients for aberration correction. Conceptual depictions, input parameters and flow diagrams are included in Supplementary Figs. S18–22. (1) The HC method is a local search algorithm inspired by mountain climbing that identifies locally (but not necessarily globally) optimal solutions (Supplementary Fig. S18)^44^. Starting at an arbitrary position in parameter space, the algorithm makes incremental steps to find the best position (as judged by the objective function, *IM*) within the neighborhood of the current position as the start for the next iteration. In our fitting procedure, optimization stops if *IM* decreases by <10^–5^ rad for ten successive iterations. Notably, HC is efficient for optimization of convex problems but cannot escape from local minima. (2) SPGD is a model-free algorithm requiring a differentiable loss function (Supplementary Fig. S19)^45^. In each iteration, stochastic perturbations are applied to the Zernike coefficients in parallel from opposite directions. By evaluating *IM*, the set of Zernike coefficients is updated for the next iteration via gradient approximation. Its parallel parameter variation enables efficient optimization in high-dimensional spaces while maintaining robustness against local minima. In our implementation, iteration stops if *IM* decreases by <10^–3^ rad for 15 successive iterations. (3) The SA algorithm was designed to find globally optimal solutions by mimicking the gradual cooling process of a heated material (Supplementary Fig. S20)^46^. In each iteration, instead of picking the best next move (as is done in HC), SA accepts or rejects a new set of parameters (Zernike coefficients) at a particular (fictive) temperature based on an exponential probability function (Boltzmann distribution), i.e., according to the Metropolis criterion^47^. The iteration process stops if *IM* decreases by <10^–5^ rad for five successive iteration rounds. (4) The PSO algorithm is a swarm intelligence algorithm that is inspired by bird flocking or fish schooling (Supplementary Fig. S21)^48^. The position of each particle represents a possible solution to the optimization problem. In the process of searching for optimal sets of parameters (Zernike coefficients), particles are initially randomly distributed and then moved around in search space according to simple rules governing their positions and velocities. The parameter updates depend on the particle’s best position (minimal loss function) and the one of the entire swarm. Eventually, this method yields a solution to the problem, yet, it cannot be guaranteed that this is the optimal solution. In our implementation, the iteration stops when the global *IM* decreases by <10^–5^ rad for five successive iterations. (5) GAs are a class of evolutionary stochastic optimization algorithms that draw inspiration from genetic principles (Supplementary Fig. S22)^49^. By using operators including selection, crossover and mutation on a population of candidates, GAs mimic natural selection. Best-fit solutions are found by combining and randomly altering individuals over multiple generations so as to minimize the loss function. In our implementation, the optimization stops if *IM* decreases by <10^–5^ rad for five successive iterations.

### Sample preparation

To prepare SPR chips, sapphire coverslips (N4247800, OLYMPUS, Japan) were thoroughly cleaned with acetone, ethanol and deionized water in an ultrasonic cleaner and blown dry with nitrogen gas. They were coated with a 1-nm Cr layer and then with a 45-nm Au layer by electron beam evaporation (Nexdep, Angstrom Engineering, USA). Afterwards, they were washed in deionized water for 10 min and subsequently in ethanol-water solvent (75:25, vol:vol) for 30 min.

To prepare EBL-structured test samples, we spin-coated an ~100 nm layer of negative photoresist (hydrogen silsesquioxane (HSQ), XR-1541, Dow Corning, USA) and then a conductive adhesive layer (~100 nm) onto the gold substrate. The designed pattern was written with EBL equipment (NB5, Nanobeam, UK). After immersion of the substrate in deionized water for ~1 min to remove the conductive adhesive, development and fixation procedures completed the EBL sample preparation.

Live cell samples were prepared as follows. Breast cancer cells (MDA-MB-231) were cultured in Dulbecco’s modified Eagle’s medium (DMEM) supplemented with 10% fetal bovine serum (FBS), 100 U mL^–1^ penicillin, and 100 μg mL^–1^ streptomycin. Cells of the murine precursor osteoblast cell line MC3T3-E1 were cultured in α-minimum essential medium (α-MEM, Gibco, USA) supplemented with 10% fetal bovine serum (FBS), 100 U mL^–1^ penicillin, and 100 μg mL^–1^ streptomycin. We seeded the cells onto the gold surface and seamlessly sealed the bottom surface with a cell chamber. The seeded cells were incubated overnight in a humidified atmosphere containing 5% CO_2_ at 37 °C. During the SPRHM measurement, the cell chamber was constantly purged with 5% CO_2_ and held at constant temperature.

### Software platform

The AO-SPRHM system is equipped with a custom software, which includes hardware control, hologram recording/reconstruction and adaptive correction functions. The fitting algorithms were run in a computer with 3.20 GHz CPU and 128 GB RAM. Except for simple user interactions such as selecting sample areas and presetting algorithm parameters, the measurement and data analysis processes were automated using our software to ensure fast and accurate aberration corrections.

## Supplementary information

Background-free quantitative phase imaging with adaptive-optics surface plasmon resonance holographic microscopy

## Data Availability

The data generated and analyzed in the article are available from the corresponding authors upon reasonable request.
