# Supplementary material for: Background-free quantitative phase imaging with adaptive-optics surface plasmon resonance holographic microscopy
Source: Light Sci Appl. 2026 Jul 14;15:317. doi: 10.1038/s41377-026-02362-x (PMC13369757; doi:10.1038/s41377-026-02362-x)
Supplement: Supplementary file 1 — Background-free quantitative phase imaging with adaptive-optics surface plasmon resonance holographic microscopy [file 41377_2026_2362_MOESM1_ESM.pdf]

Supplementary Information for:

Background-free quantitative phase imaging with adaptive-optics surface plasmon resonance holographic microscopy

Siqing Dai[1], Mengmeng Zhang[1], Yushan Shen[2], Haoyu Xu[1], Li Ren[2], Hua Lu[1], Jiwei Zhang[1*], Gerd Ulrich Nienhaus[3,4,5*], and Jianlin Zhao[1*]

[1]*Key Laboratory of Light Field Manipulation and Information Acquisition, Ministry of Industry and Information Technology, and Shaanxi Key Laboratory of Optical Information Technology, School of Physical Science and Technology, Northwestern Polytechnical University, Xi'an, 710129, China*
[2]*Key Laboratory for Space Bioscience and Biotechnology, School of Life Science, Northwestern Polytechnical University, Xi'an, 710072, China*
[3]*Institute of Applied Physics, Karlsruhe Institute of Technology, 76131 Karlsruhe, Germany*
[4]*Institute of Biological and Chemical Systems and Institute of Nanotechnology, Karlsruhe Institute of Technology, 76344 Eggenstein-Leopoldshafen, Germany*
[5]*Department of Physics, University of Illinois at Urbana-Champaign, Urbana, Illinois 61801, United States*

Email addresses of the corresponding authors:

[*]*jwzhang@nwpu.edu.cn;*

[*]*uli@uiuc.edu;*

[*]*jlzhao@nwpu.edu.cn.*

Supplementary Notes

Note 1: Hologram recording and reconstruction

1. Hologram recording

In the hologram recording plane, the complex amplitude distributions of the object wave, $O(x, y)$, and the reference wave, $R(x, y)$, can be written as

$$\begin{aligned} O(x,y) &= A_O(x,y)\exp[\mathrm{i}\varphi_O(x,y)], \\ R(x,y) &= A_R(x,y)\exp[\mathrm{i}\varphi_R(x,y)], \end{aligned} \tag{S1}$$

respectively, where $A_O(x, y)$ ($A_R(x, y)$) and $\varphi_O(x, y)$ ($\varphi_R(x, y)$) are the amplitudes and phases of the object (reference) wave, respectively. The object and reference waves interfere in the overlap region, yielding an intensity distribution

$$\begin{aligned} I(x,y) &= |O(x,y)+R(x,y)|^2 \\ &= |O(x,y)|^2+|R(x,y)|^2+O(x,y)R^*(x,y)+O^*(x,y)R(x,y) \\ &= A_O^2(x,y)+A_R^2(x,y)+2A_O(x,y)A_R(x,y)\cos[\varphi_O(x,y)-\varphi_R(x,y)]. \end{aligned} \tag{S2}$$

2. Hologram reconstruction

In traditional optical holography, the same reference wave illuminates the interference pattern on the recording plate, so the complex amplitude of the transmitted light wave is given by

$$\begin{aligned} u(x,y) &= R(x,y)I(x,y) \\ &= |O(x,y)|^2R(x,y)+|R(x,y)|^2R(x,y)+|R(x,y)|^2O(x,y)+R(x,y)^2O^*(x,y) \\ &= u_1+u_2+u_3+u_4. \end{aligned} \tag{S3}$$

Notably, u_3 is the complex amplitude of the recovered object wave multiplied by a constant. In off-axis holography, u_3 and u_4 are well separated from u_1 and u_2 and can thus be easily measured. In digital holography, the hologram is recorded by a CCD camera, and the reconstruction of the object wave is performed by a computer. Here, we utilize the convolution method for numerical reconstruction, which analyzes light wave propagation in the Fourier domain[1, 2]. Specifically, treating the complex amplitude distribution of the light wave in the hologram plane as a coherent superposition of plane waves propagating in different directions, the

convolution method regards the complex amplitude distribution of the light wave in the image plane as a linear combination of plane waves with phase shifts. The magnitude of the phase shift of the plane waves depends on the optical transfer function (OTF), i.e., the Fourier transform of the point spread function (impulse response) of the optical system.

The complex amplitude of the light in the image plane (coordinates ξ, η) at a distance, d, from the hologram plane (coordinates x, y) is given by

$$U_d(\xi,\eta)=\int_{-\infty}^{\infty}\int_{-\infty}^{\infty}R(x,y)I(x,y)h(\xi,\eta,x,y)\mathrm{d}x\mathrm{d}y \quad \text{(S4)}$$

where $h(\xi, \eta, x, y,)$ is the impulse response function in the paraxial approximation,

$$h(\xi,\eta,x,y)=\frac{\mathrm{i}}{\lambda}\frac{\exp[-\mathrm{i}\frac{2\pi}{\lambda}\sqrt{d^2+(\xi-x)^2+(\eta-y)^2}]}{\sqrt{d^2+(\xi-x)^2+(\eta-y)^2}}, \quad \text{(S5)}$$

and λ is the wavelength of the light.

According to the convolution method[1, 2], Eq. S4 can also be written as

$$\begin{aligned}U_d(\xi,\eta)&=F^{-1}\{F[R(x,y)I(x,y)]\cdot F[h(\xi,\eta,x,y)]\}\\&=F^{-1}\{F[R(x,y)I(x,y)]\cdot H\}\end{aligned}, \quad \text{(S6)}$$

with H representing the discretized OTF,

$$H(x,y)=\exp\left[\mathrm{i}\frac{2\pi d}{\lambda}\left(1-\frac{\lambda^2}{X^2\Delta x^2}\left(x+\frac{X^2\Delta x^2}{2d\lambda}\right)^2-\frac{\lambda^2}{Y^2\Delta y^2}\left(y+\frac{Y^2\Delta y^2}{2d\lambda}\right)^2\right)^{1/2}\right].$$

(S7)

Here, X and Y denote the pixel numbers of the CCD camera in the horizontal and vertical directions, respectively, and the corresponding pixel sizes are Δx and Δy, respectively, x = 1, 2, … X, y = 1, 2, … Y.

In practice, the reference wave is not a priori known. Rather, a plane wave is taken in the reconstruction, so that Eq. S6 simplifies to

$$U_d(\xi,\eta)=F^{-1}\{F[I(x,y)]\cdot H\} \quad \text{(S8)}$$

Accordingly, the object wave is calculated by first taking the fast Fourier transform of the digital hologram. After extracting the ± first orders of the Fourier spectrum and multiplying with H, the inverse Fourier transform finally yields the object wave distribution. The intensity and phase distributions, $I(\xi, \eta)$ and $\varphi(\xi, \eta)$, of the object wave can be calculated by

$$\begin{cases} I(\xi,\eta) = |U_d(\xi,\eta)|^2 \\ \varphi(\xi,\eta) = \arctan\left\{\dfrac{\mathrm{Im}[U_d(\xi,\eta)]}{\mathrm{Re}[U_d(\xi,\eta)]}\right\} \end{cases}, \tag{S9}$$

in which $\mathrm{Re}[U_d(\xi, \eta)]$ and $\mathrm{Im}[U_d(\xi, \eta)]$ are the real and imaginary parts of the complex amplitude of the object wave, respectively. Notably, the image plane holography was used in this work, with the reconstruction distance $d = 0$. Thus, the hologram plane and image plane are actually the same plane.

Importantly, the reference wave is not an ideal plane wave in practice. As a result, the phase distribution reconstructed from the hologram is the phase difference between the object and reference waves, and not the phase of the object wave itself. Therefore, the phase image measured by SPRHM is

$$\begin{aligned} \varphi(x,y) &= \varphi_O(x,y) - \varphi_R(x,y) \\ &= \varphi_{\mathrm{SPR}}(x,y) - \varphi_R(x,y) \\ &= \varphi_{\mathrm{s}}(x,y) + \varphi_{\mathrm{a}}(x,y) - \varphi_R(x,y), \end{aligned} \tag{S10}$$

where we substituted $\varphi_{\mathrm{SPR}}(x, y)$ using Eq. 1 in the main text. Accordingly, the phase pattern that we need to upload to the SLM to compensate aberrations is

$$\varphi_{\mathrm{m}}(x,y) = \varphi_R(x,y) - \varphi_{\mathrm{a}}(x,y). \tag{S11}$$

When the wavefront of the object wave is modulated by the SLM phase pattern, the phase difference between object and reference waves of the digital hologram is given by

$$\left.\begin{aligned} \varphi'(x,y) &= \varphi_O'(x,y) - \varphi_R'(x,y), \\ \varphi_O'(x,y) &= \varphi_{\mathrm{s}}(x,y) + \varphi_{\mathrm{a}}(x,y) + \varphi_{\mathrm{m}}(x,y) \\ &= \varphi_{\mathrm{s}}(x,y) + \varphi_R(x,y), \\ \varphi_R'(x,y) &= \varphi_R(x,y). \end{aligned}\right\} \rightarrow \varphi'(x,y) = \varphi_{\mathrm{s}}(x,y). \tag{S12}$$

Therefore, after AO correction, the numerically reconstructed phase distribution is the correct SPR phase image of the sample. Note that this conclusion only holds true if the reference beam is not phase-modulated by the SLM.

Note 2: Mapping the phase to the cell adhesion gap width based on a six-layer SPR model

Under SPR excitation, the surface plasmon polariton propagates along the interface, with its intensity perpendicular to the gold surface decaying exponentially, with a characteristic length of 100 – 200 nm at the HeNe laser wavelength of 632.8 nm. Consequently, the extension of the evanescent wave is small against the overall thickness of adherent cells (typically several micrometer) yet perfectly matched to the thickness of the adhesion cleft. To map the phase shift to a gap thickness, we use the SPR model sketched in Fig. S3a, which includes six layers, (1) coverslip, (2) Cr layer, (3) Au layer, (4) cell adhesion gap containing culturing medium in varying thickness, (5) cell membrane and (6) cytoplasm. For this model, we calculate the reflection coefficient of the light wave as a function of the cell adhesion gap according to Fresnel's equations[3]. Notably, all physical parameters (thickness and dielectric constant) are known for each layer except for the gap thickness[4]. The resulting dependence of the SPR phase, obtained from the complex reflection coefficient, on the cell adhesion gap is plotted in Fig. S3b. This monotonically decaying curve allows us to convert the measured phases into the adhesion gap for each pixel, as described in our previous work[4].

Note 3: Background area needed for proper aberration correction in cell measurements

To investigate the amount of background area needed for precise correction of aberrations, we superimposed a concentric circular mask of zero phase inside with the original distorted phase images of the acquired cell samples. By changing the radius of the circular mask, we adjusted the area that was used as background for aberration correction. For quantification, we define an area ratio as the fraction of background pixels,

$$R_{\mathrm{BG}}=\frac{r_2^{\,2}-r_1^{\,2}}{r_2^{\,2}}, \tag{S13}$$

where r_2 is the full radius of Zernike modes, and r_1 is a radius varying between 0 and r_2, as depicted in Fig. S10, using the image in Fig. 3 (main text) as an example. Thus, R_{BG} varies between 1 (no mask, largest background region) and 0 (largest mask, no background region).

We employed the image metric *IM* (Eq. 4 in the manuscript) to quantify the adaptive correction performance as a function of R_{BG}. Specifically, the distorted phase images were first masked for different R_{BG} values, and then corrected with the HC algorithm. The corrected SPR phase images were subsequently processed with a circular mask which precisely excludes the effects of the phase in the cellular region, and *IM* was calculated.

Figure S11 shows the corrected SPR phase images (again based on Fig. 3) for the original aberrant phase images superimposed with circular masks of various size. For R_{BG} decreasing from 0.9 to 0 in steps of 0.1, the corrected images degrade markedly, as is also seen from the increasing *IM*.

We further extended our analysis to 16 live cell samples. The average *IM* for R_{BG} in the range 0 and 1 in steps of 0.05 is plotted in Fig. S12, showing the expected image quality deterioration with decreasing background area. If we demand that *IM* should not increase by $\geq 10^{-3}$ rad, a minimum R_{BG} of ~0.35 can be a deduced from an exponential fit (Fig. S12).

Notably, the circular mask approach works well for our samples, where the cells are compact, isolated and located in the center of the field of view, but not in general. Also, it will be less effective if aberrations with higher spatial frequency are present, requiring higher-order Zernike modes for AO correction.

Note 4: Control experiment with artificially introduced coma in the SPRHM and adaptive correction with and without coma Zernike modes

Human breast cancer cells were imaged with coma in the SPRHM system, which was artificially introduced by using the SLM. In the first round, horizontal coma (coefficient = 1) was included, and the adaptive correction (HC algorithm) was carried out using the first six (without coma) and then eight (including horizontal and vertical coma) Zernike modes. In the second round, vertical coma (coefficient = 1) was examined in the same way.

Our data (Fig. S13) reveal the obvious, i.e., that aberrations can only be fully compensated if the associated Zernike modes are accounted for. To quantify the reconstruction errors, we calculated the *IM* of the corrected images by adding a circular mask (R_{BG} = 1) to remove the sample region (Table S3). These data indicate that *IM* is significantly greater if higher modes are present in the system, yet only the first six Zernike modes are included in the AO process.

Note 5: L_1 and L_2 regularization in the fitting process

The L_1 loss function is defined as the sum of absolute differences between target values y_i and the corresponding estimates, $f(x_i)$, while the L_2 loss function, also known as least squares error (LSE), minimizes the sum of the squared differences between y_i and $f(x_i)$,

$$
\begin{aligned}
L_1 &= \sum_{i=1}^{n} \left| y_i - f(x_i) \right|, \\
L_2 &= \sum_{i=1}^{n} \left[y_i - f(x_i) \right]^2.
\end{aligned}
\tag{S14}
$$

L_1 regularization confers robustness to outliers by imposing linear penalties on residuals; L_2 regularization imposes severe penalties on weight factors with large numerical values. In our case, overfitting the sparse background results in anomalously large Zernike coefficients. With relatively smooth and low-order background aberrations in the SPRHM system, the Zernike coefficients should be reasonably small. Thus, Eq. S14 can be approximated by

$$
\begin{aligned}
L_1 &\approx \sum_{i=1}^{n} \left| C_i \right|, \\
L_2 &\approx \sum_{i=1}^{n} C_i^2,
\end{aligned}
\tag{S15}
$$

with C_i being the coefficient of the ith Zernike mode. We added the L_1/L_2 functions to the original objective function, the image metric *IM*,

$$
Loss = IM + kL_j, \ (j = 1, 2), \tag{S16}
$$

where k is the weighting factor. For an aberrant SPR phase image (Fig. 3 in the main text), we implemented the AO correction via the HC algorithm with the new *Loss* function as the objective function. To determine the weighting factor k, we gradually increased its value from 0 to 1 with the step of 0.01 and performed the AO corrections based on the *Loss* function for >10 distorted phase images of cell samples using the HC method. The corrected phase images were then evaluated with *IM* after superimposing a circular mask to remove the sample region. The results of *IM* as a function of the weighting factor k are shown in Fig. S14a (for L_1 regularization) and Fig. S14b (for L_2 regularization). Clearly, the correction works poorly for larger k. Quantitatively, for L_1, *IM* is low and comparable to that based on original objective function up to $k = 0.1$. For L_2, the *IM* becomes greater than with the original objective function for $k > 0.01$. Consequently, we set the factor k to be 0.1 and 0.01 for L_1 and L_2 in the *Loss*

function, respectively. The results are compared with our prior treatment in Fig. S15. Notably, the SPR images corrected by using *Loss* (Figs. S15d and S15e) are similar to the one by the *IM* metric (Fig. S15f). In quantitative terms, *IM* values of the corrected images superimposed with a circular mask based on *Loss* and the original *IM* are 0.37 rad (L_1), 0.35 rad (L_2) and 0.35 rad, respectively.

Supplementary figures

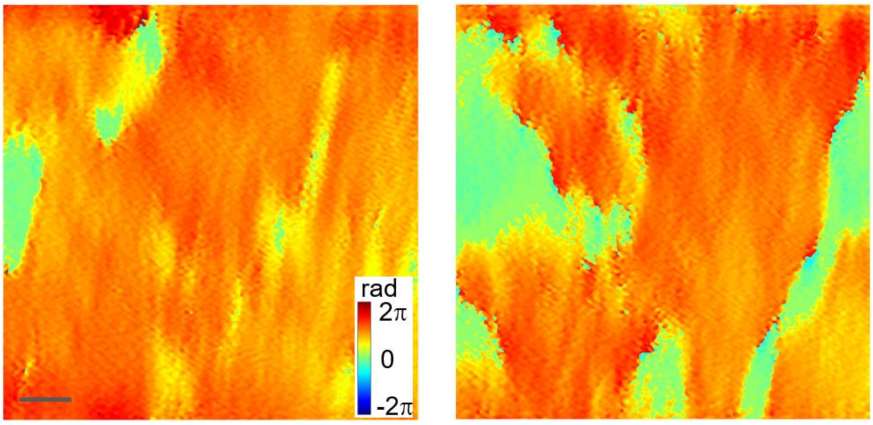

Fig. S1. Two examples of background-corrected SPR phase images of dense layers of breast cancer cells. AO-SPRHM with HC optimization was employed for correction. Scale bar: 5 μm.

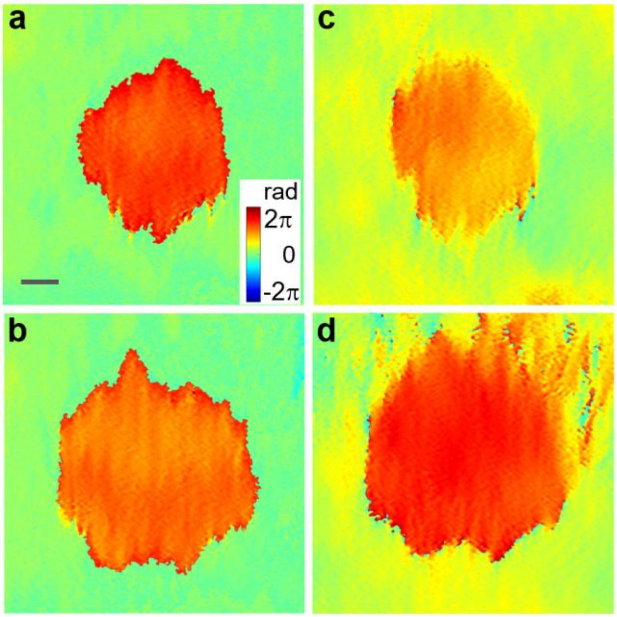

Fig. S2. SPR phase images of mouse osteoblast cells, background-corrected with different numbers of Zernike modes. a, b Two exemplary images, with background corrected by using the first six Zernike modes (piston, x- and y-tilt, defocus and oblique/vertical astigmatism). **c, d** Alternative background correction with the first eleven Zernike modes (including, in addition, oblique/vertical coma, oblique/vertical trefoil and primary spherical aberration). The HC algorithm was employed for optimizing the adaptive correction. Scale bar: 5 μm.

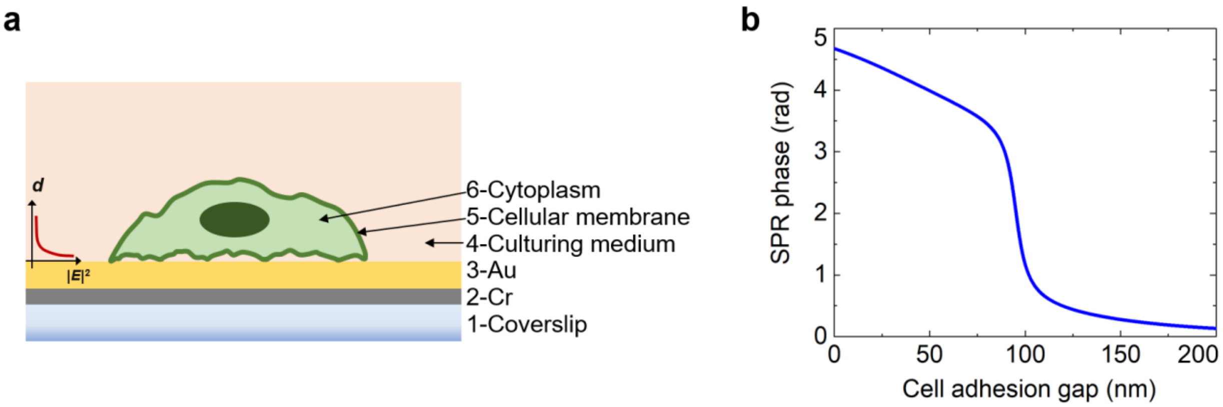

Fig. S3. Mapping the phases of the measured phase image to the cell adhesion gap. a Six-layer SPR model, *E*: electric field, *d*: penetration depth of the evanescent wave. **b** Dependence of the SPR phase on the cell adhesion gap, as calculated with the SPR model.

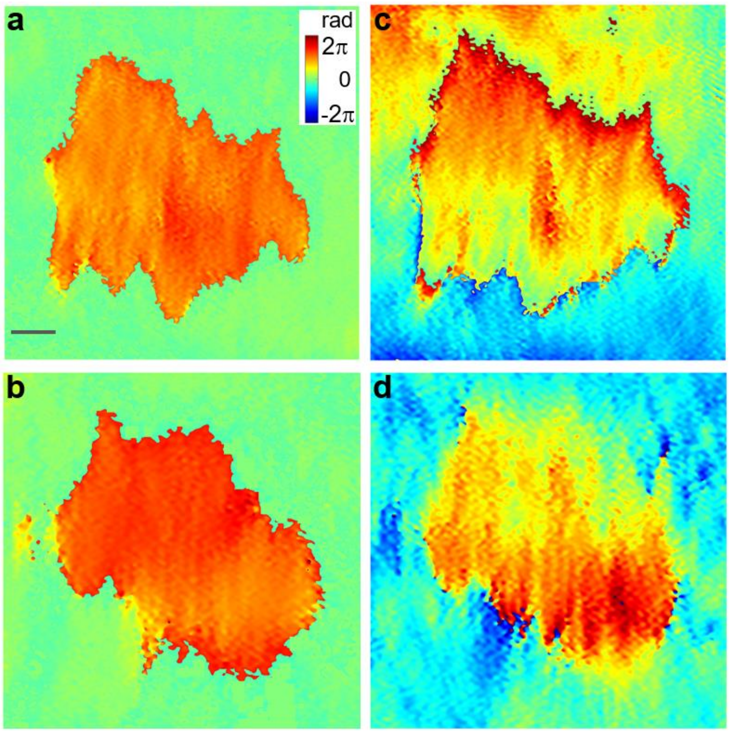

Fig. S4. Exemplary background-corrected SPR phase images of two breast cancer cells. a, b AO-SPRHM with HC optimization. **c, d** DE method. Scale bar: 5 µm.

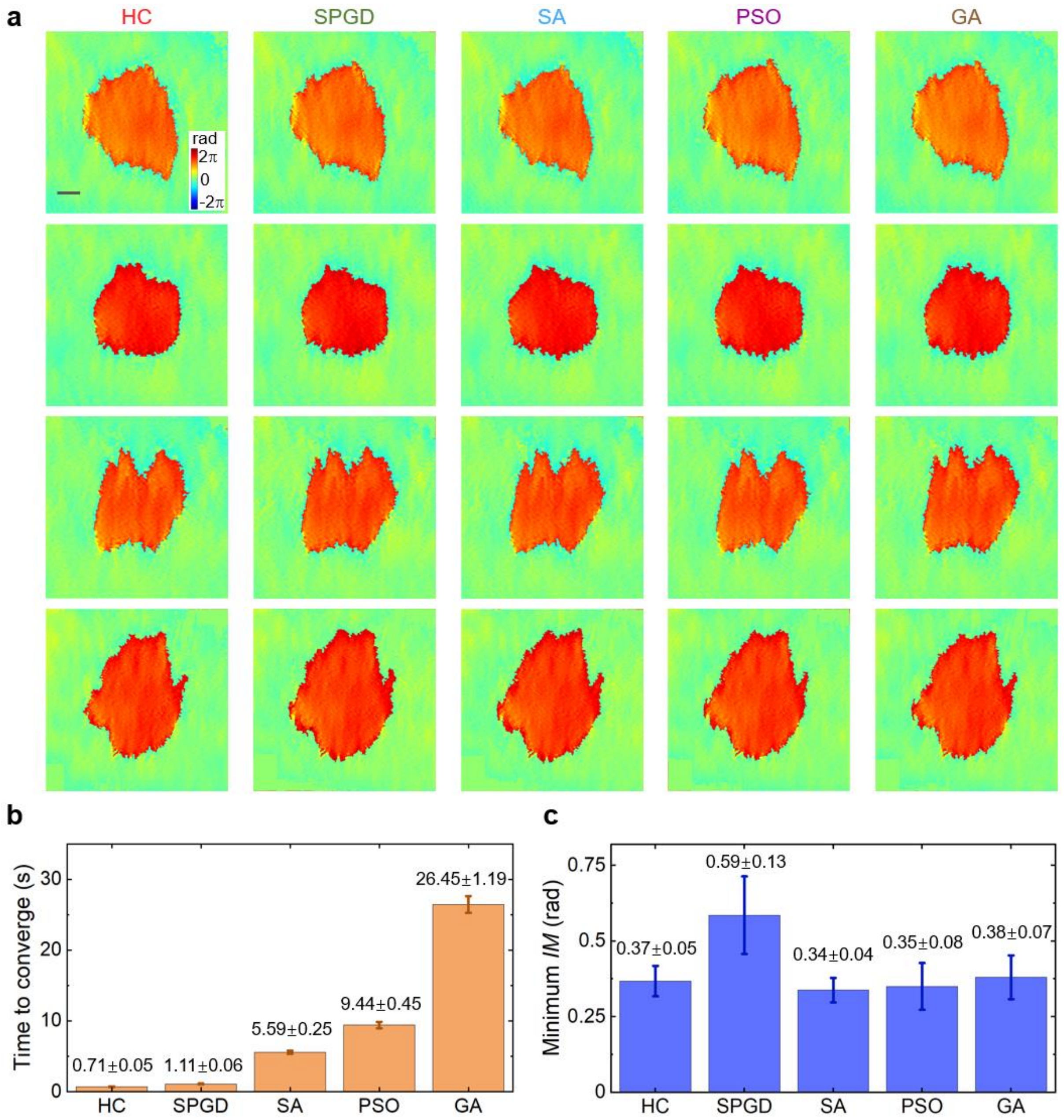

Fig. S5. Statistical comparison of optimization algorithm performances. a Exemplary aberration-corrected SPR phase images of four breast cancer cells. **b** Time to convergence and **c** minimum *IM* (after convergence) for the five algorithms employed. For correction speed, fitting is the most time-consuming step and can serve well as a measure of the overall speed of background correction. The value of *IM* after convergence is regarded as a measure of the correction accuracy. For each cell, the algorithms were employed one after another but in random order to randomize variations in computer speed and memory usage. From **b** and **c**, it can be seen that HC outperforms the other optimization methods with a correction speed of 0.71 s and an accuracy of 0.37 rad. Scale bar: 5 μm.

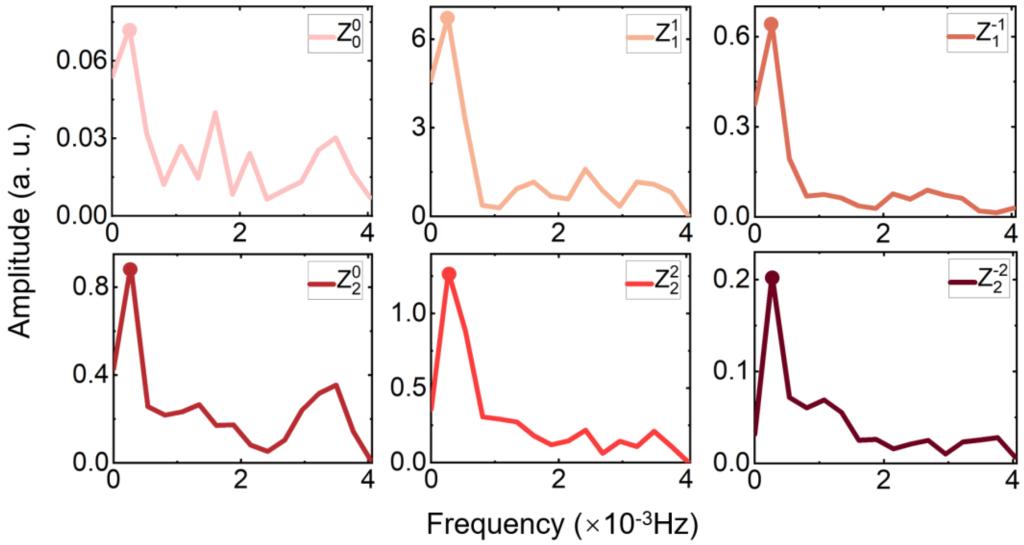

Fig. S6. Frequency spectra of the Zernike mode fluctuations shown in Fig. 5h in the main text, calculated by Fast Fourier Transformation (FFT).

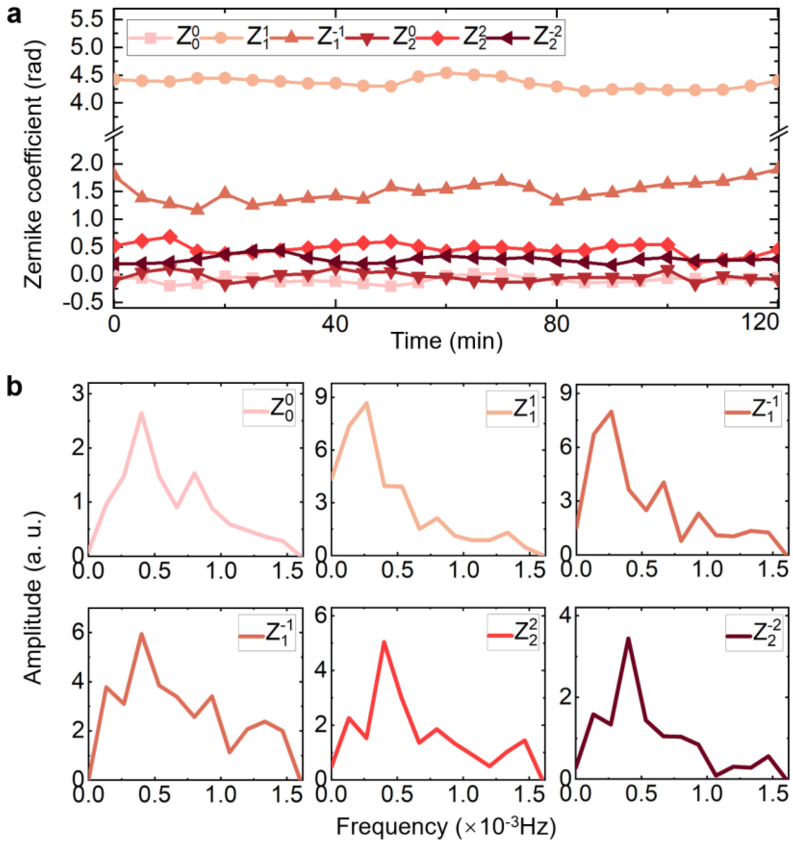

Fig. S7. Continuous aberration correction experiment with a correction time interval of 5 min over 2 h. **a** Temporal variation of the measured Zernike coefficients, **b** frequency spectra of the fluctuations calculated by FFT.

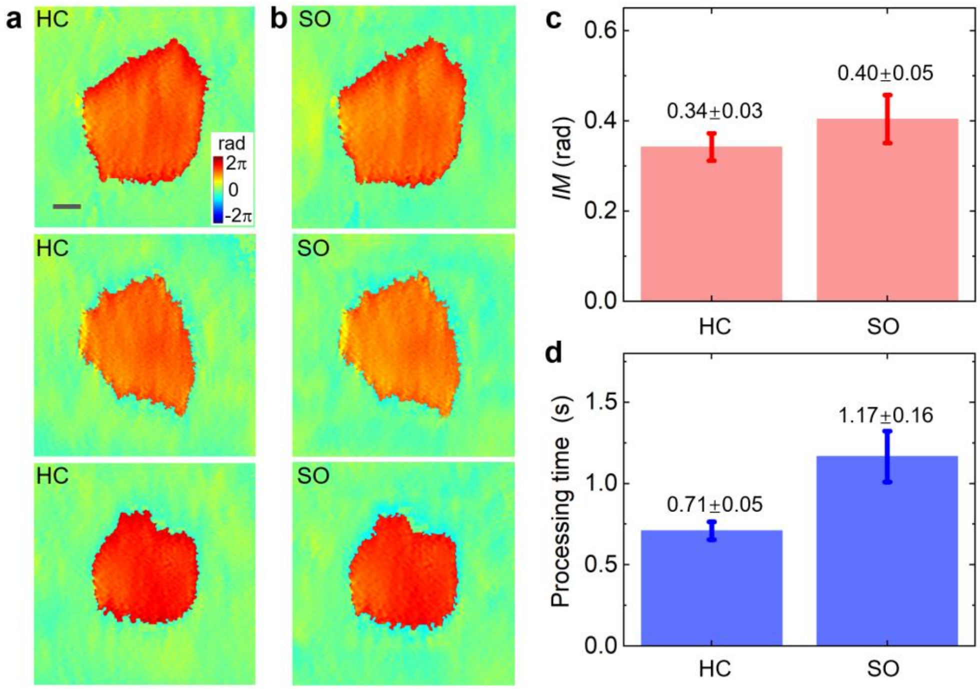

Fig. S8. Comparison of aberration correction performances between AO-SPRHM and the sparse optimization (SO) method[5]. Exemplary corrected SPR phase images of cell samples using **a** HC and **b** SO. Comparisons between SO and HC of **c** image metric (*IM*) and **d** processing time. Heights of the bars, averages over 10 cell images; error bars, standard deviation. Scale bar: 5 μm.

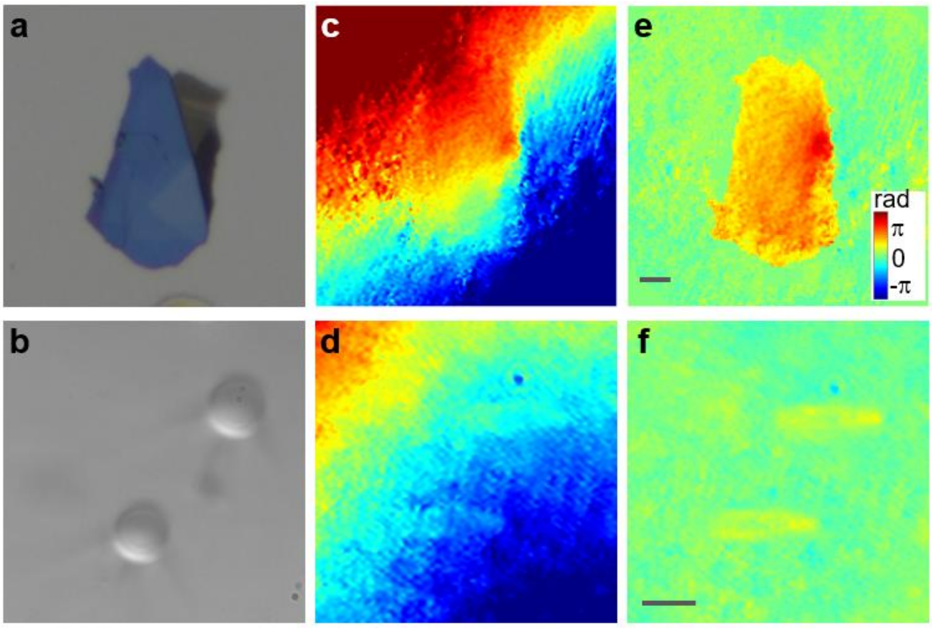

Fig. S9. SPR phase imaging of diverse samples by regular SPRHM and AO-SPRHM. Optical images of **a** graphene flakes and **b** K562 cell samples; **c, d** the corresponding original aberrant SPR images and **e, f** aberration-corrected SPR images. Scale bar, 5 μm. Sample preparation: graphene flakes were transferred to the gold substrate by mechanical exfoliation. Suspended K562 cells were cultured as previously described[6]. The cells were dropped into the evanescent field area and detected by SPRHM. Of note, the phase pattern of the suspended K562 cells is elongated along the horizontal direction due to surface plasmon wave propagation.

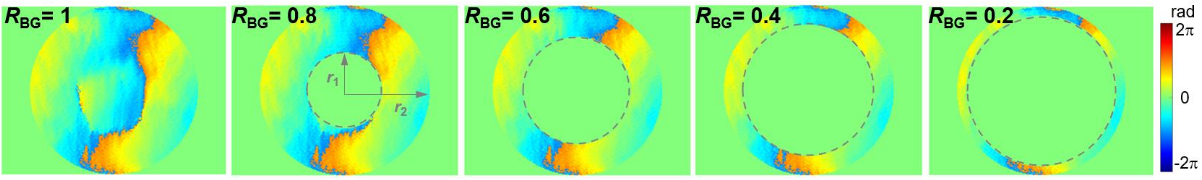

Fig. S10. Aberrant SPR phase image (Fig. 3 in the main text), masked by circular regions (phase = 0) of various sizes, yielding the indicated background ratios, R_{BG}.

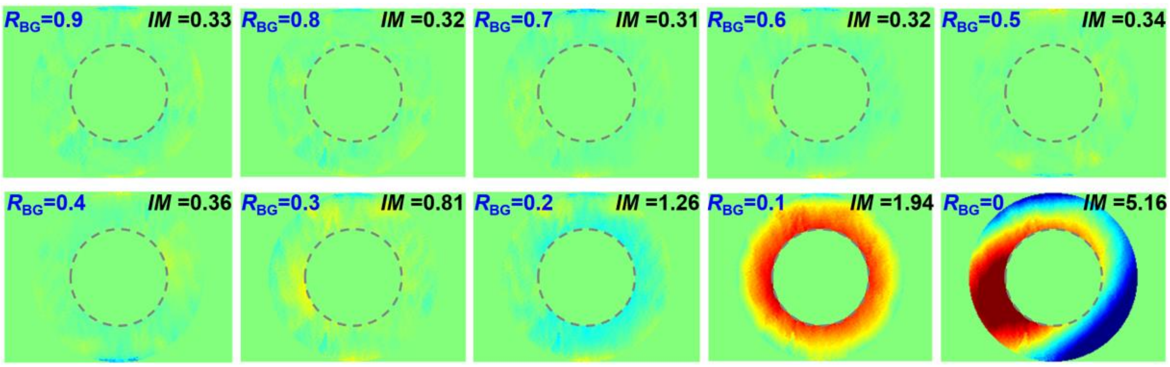

Fig. S11. Masked corrected SPR phase images with AO background ratio R_{BG} decreasing from 0.9 to 0 in steps of 0.1. *IM*: image metric.

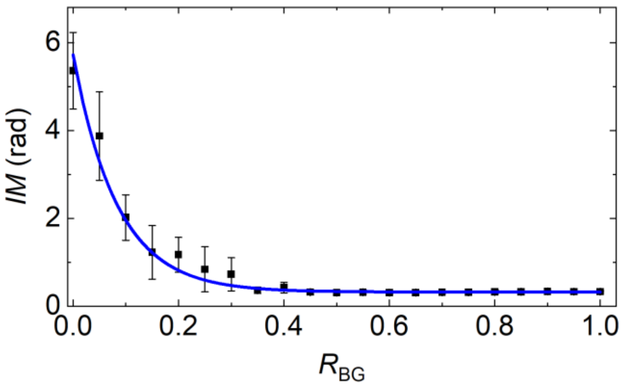

Fig. S12. Statistics of *IM* for background phase correction with a circular mask as a function of R_{BG}. Symbols, averages over 16 cell images; error bars, standard deviation; line, fit with a decaying exponential function (plus offset).

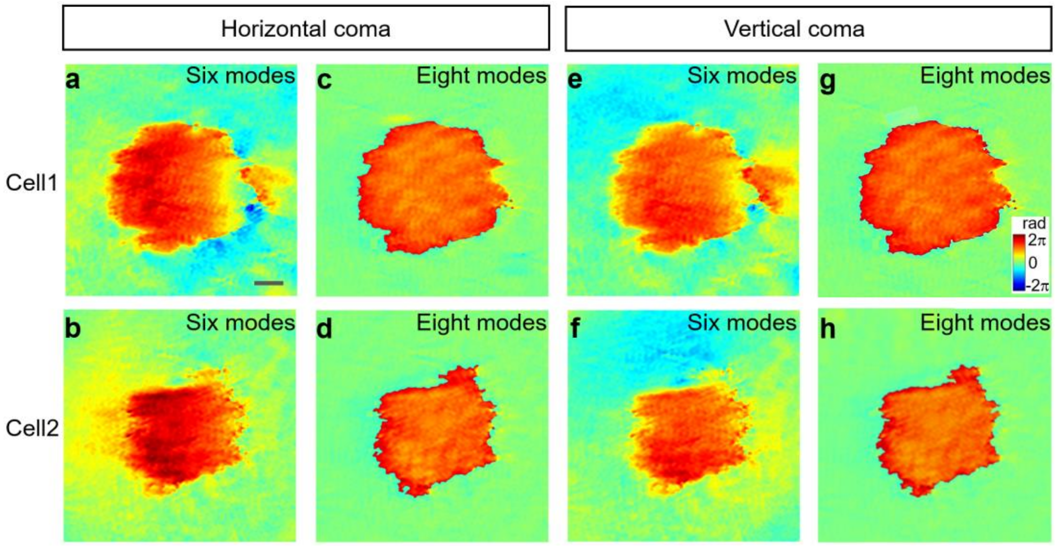

Fig. S13. Two examples of cell measurements to examine the effects of coma in the SPRHM system. SPR images with artificially applied horizontal coma (coefficient = 1), corrected with the first **a**, **b** six Zernike modes (without coma) and **c**, **d** eight Zernike modes (including coma). SPR images with artificially applied vertical coma (coefficient = 1), corrected with the first **e**, **f** six Zernike modes (without coma) and **g**, **h** eight Zernike modes (with coma). Scale bar, 5 μm.

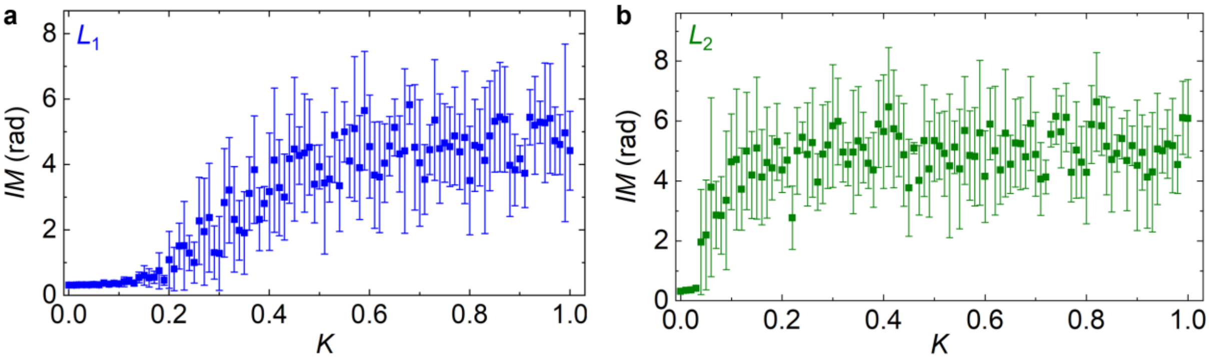

Fig. S14. Image metric (*IM*) as a function of weighting factor *k* in the *Loss* function (Eq. S16) with **a** L_1 and **b** L_2 regularization. Symbols, averages over ten cell images; error bars, standard deviation.

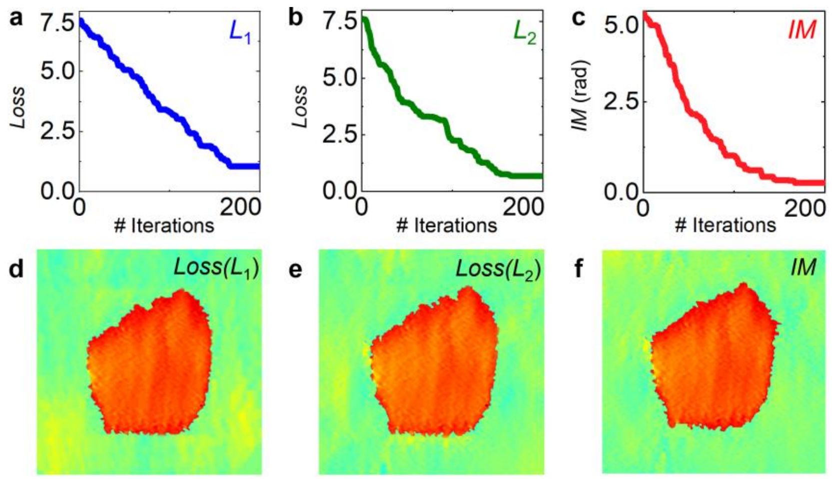

Fig. S15. Comparison of AO correction between the *Loss* function and image metric *IM* using the HC method. Minimization of **a** *Loss* (L_1), **b** *Loss* (L_2) and **c** *IM* as a function of the number of iterations. Aberration-free SPR phase images when using **d** *Loss* (L_1), **e** *Loss* (L_2) and **f** *IM* as the objective function.

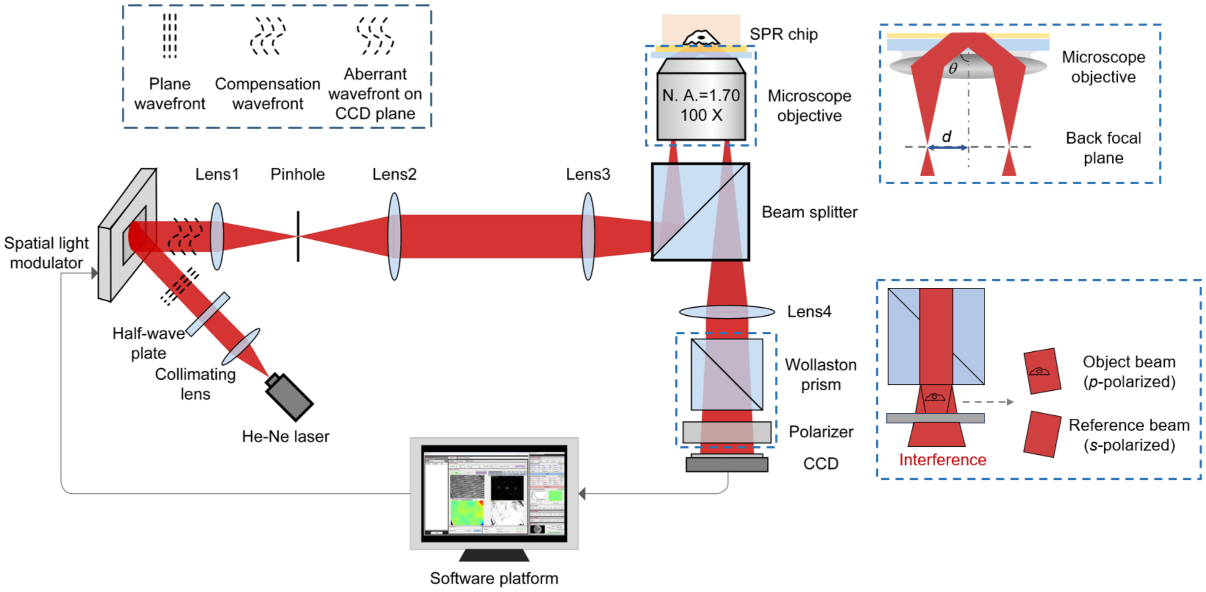

Fig. S16. Optical setup of AO-SPRHM. A HeNe laser beam, linearly polarized at 45°, is reflected off a phase-only spatial light modulator (SLM). Importantly, only the *p*-polarized component is phase-modulated and diffracted into the first order, the *s*-polarized component only reflected. Both components pass a pinhole positioned such that higher orders of the *p*-polarized component are rejected. The beam illuminates the SPR chip. Since SPR can only be excited by the *p*-polarized component, this component carries the sample information and so serves as the object beam, and the unmodulated *s*-polarized beam is the reference beam. The two components propagate on a common path up to the Wollaton prism, which splits them with a small angle (~2.1° at 632.8 nm). After the polarizer at 45°, the two beams have the same linear polarization and interfere to generate an off-axis hologram. Compared to traditional interferometers using different pathways, the common-path structure minimizes mechanical instability of the imaging system.

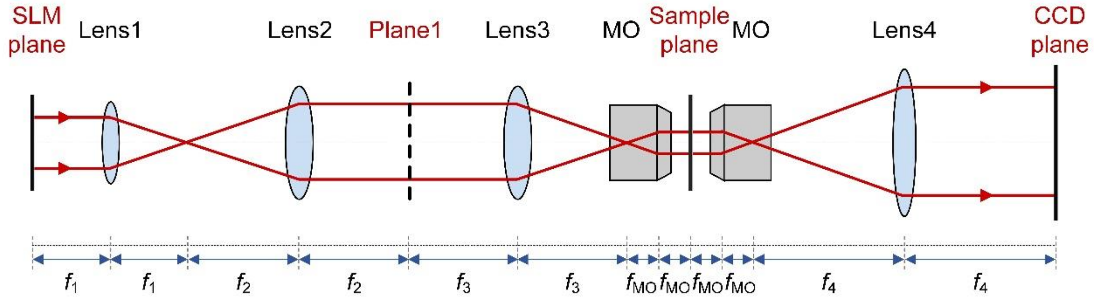

Fig. S17. Imaging path of our AO-SPRHM system with three telescopes in sequence. In the first 4*f* system consisting of Lens1 and Lens 2, the SLM plane is conjugated to Plane 1. This plane is imaged onto the sample plane by the second 4*f* system consisting of Lens3 and microscope objective (MO). The third 4*f* system consisting of MO and Lens 4 conjugates the sample plane to the CCD plane.

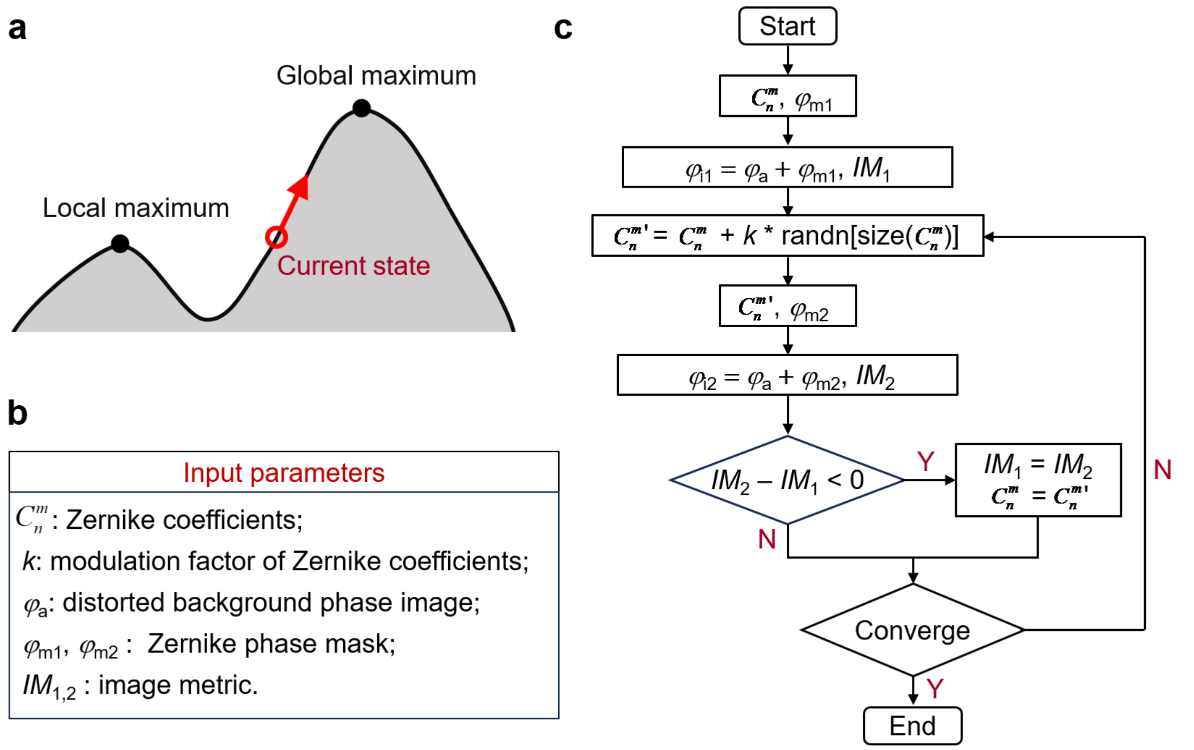

Fig. S18. HC algorithm for Zernike coefficient determination. a Illustration of the principle of the algorithm. **b** Input parameters. **c** Flow chart. randn: a random value drawn from a Gaussian distribution ($\mu = 0$; $\sigma = 1$). Since the loss function was minimized in this work, we employed this method to search for a minimum rather than a hilltop.

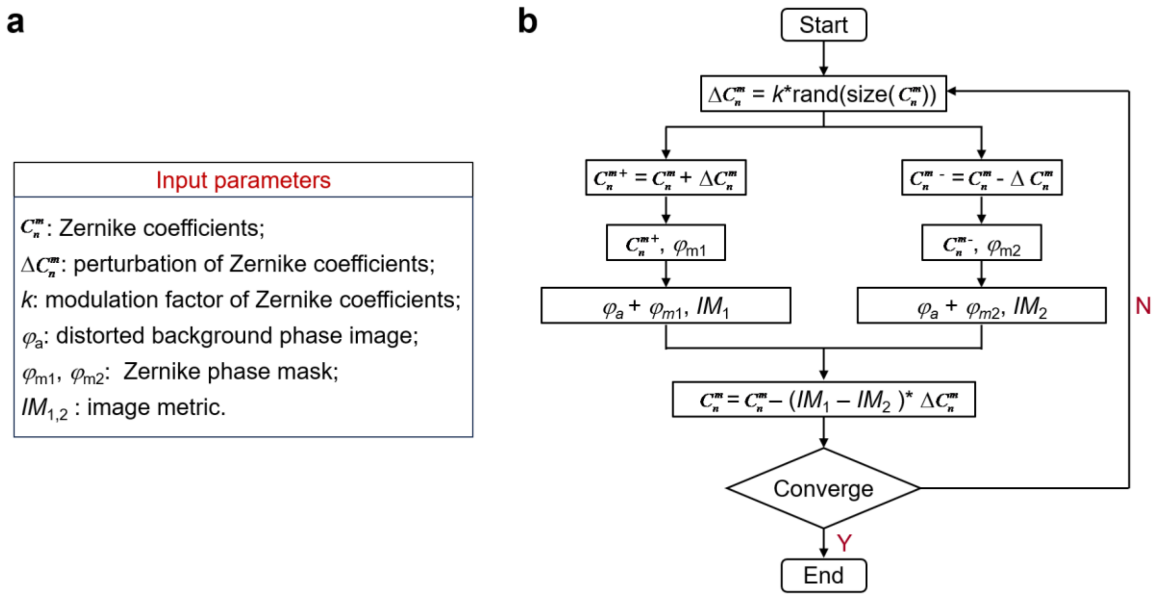

Fig. S19. SPGD algorithm for Zernike coefficient determination. a Input parameters. **b** Flow chart. rand: a random value evenly distributed in [0, 1].

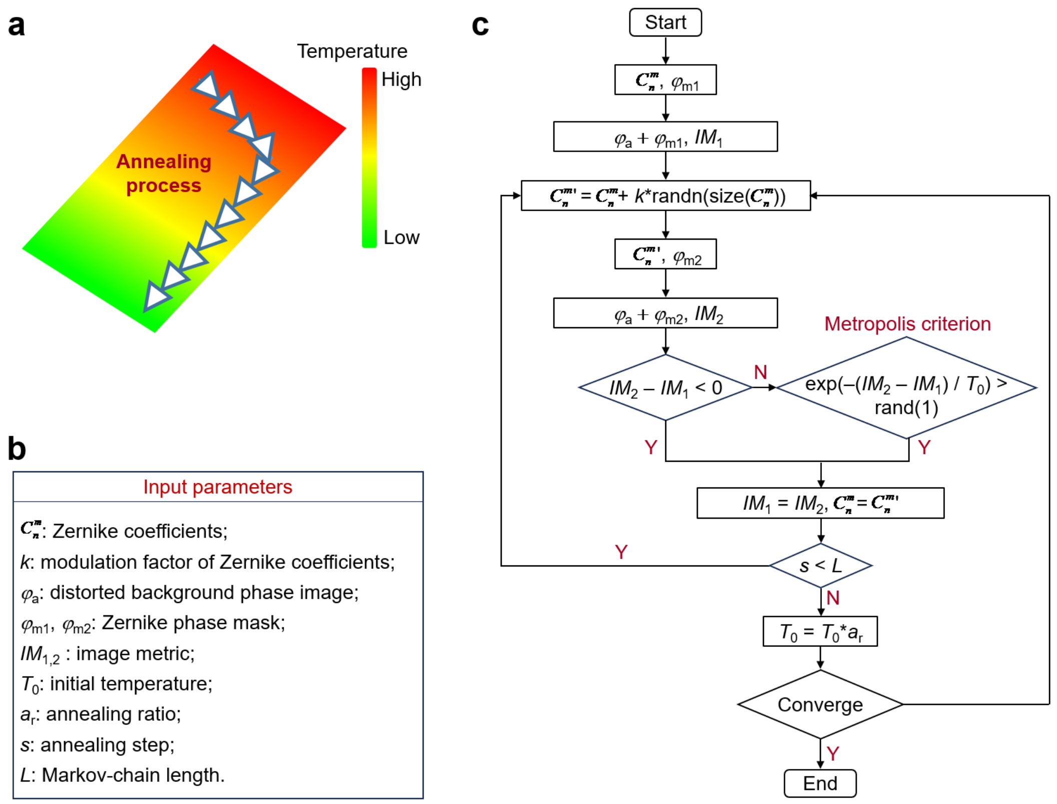

Fig. S20. SA algorithm for Zernike coefficient determination. a Illustration of the principle of the algorithm. **b** Input parameters. **c** Flow chart.

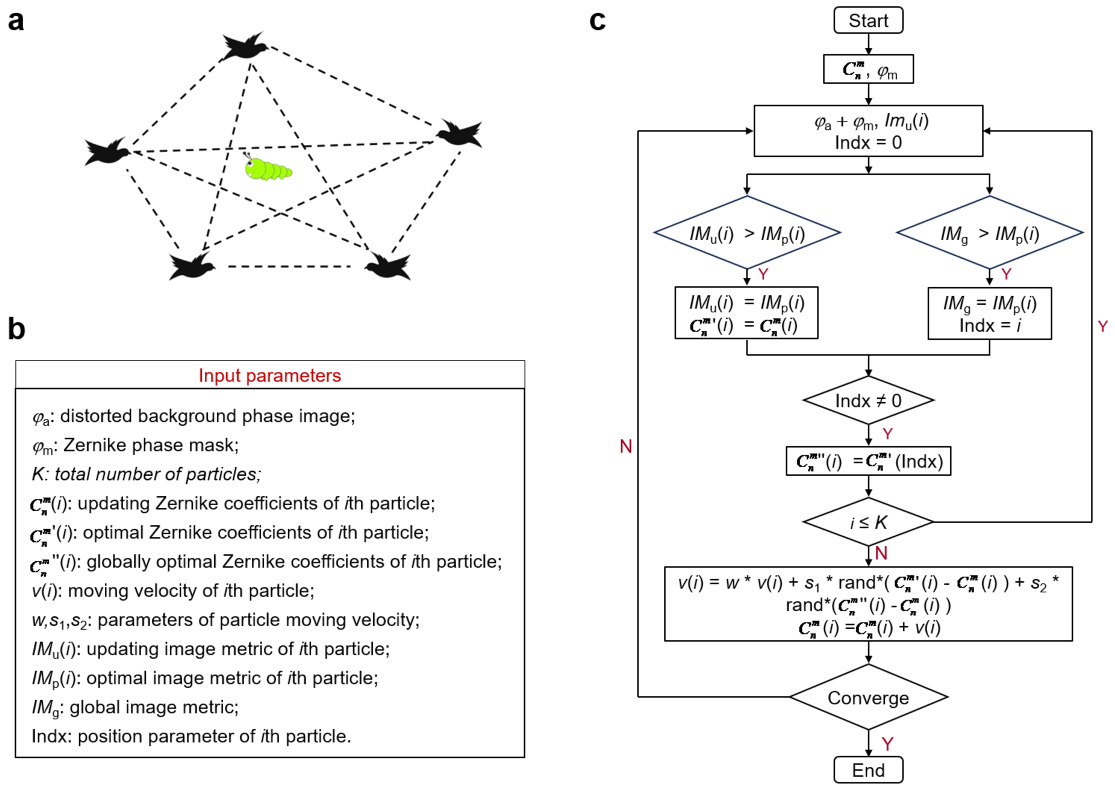

Fig. S21. PSO algorithm for Zernike coefficient determination. a Illustration of the principle of the algorithm. **b** Input parameters. **c** Flow chart.

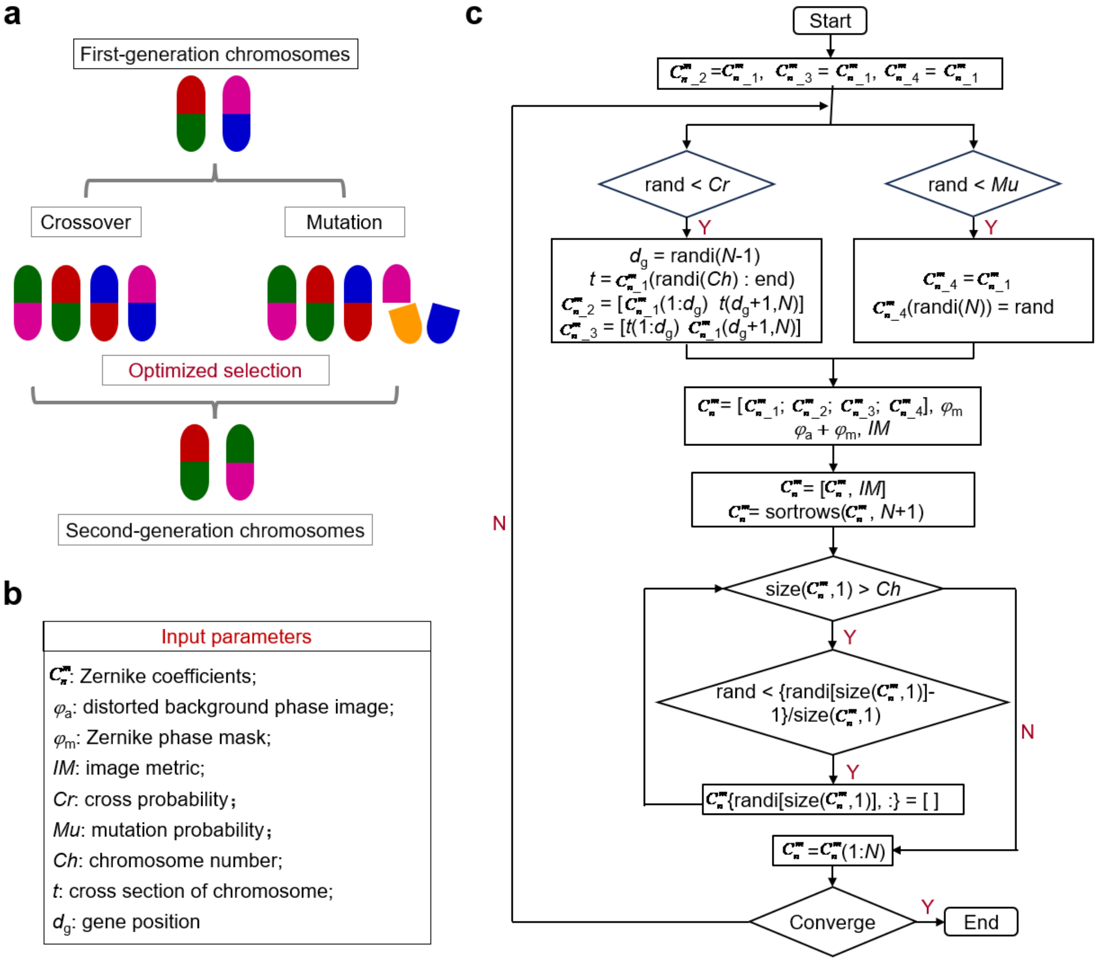

Fig. S22. GA algorithm for Zernike coefficient determination. a Illustration of the principle of the algorithm. **b** Input parameters. **c** Flow chart. randi(N): pseudo-random integer evenly distributed in [0, N].

Supplementary Tables

Table S1. Best-fit Zernike coefficients from the five algorithms

Zernike coeff. (rad) / Algorithm	C_0^0	C_1^1	C_1^{-1}	C_2^0	C_2^2	C_2^{-2}
HC	–0.11	3.64	5.08	0.15	–0.07	0.24
SPGD	–0.13	4.07	4.66	0.43	0.28	0.40
SA	–0.08	3.84	4.87	0.29	0.24	0.37
PSO	–0.12	3.90	4.81	0.37	0.23	0.42
GA	0.00	3.50	4.83	0.15	0.27	0.26

Table S2. Background parameters of SPR phase images for different algorithms

Correction method / Parameter	DE	HC	SPGD	SA	PSO	GA
Mean value (rad)[1]	0.04	–0.05	–0.03	–0.04	–0.05	–0.03
Standard deviation (rad)[1]	0.18	0.18	0.18	0.19	0.18	0.19

[1] as calculated from the background region of the SPR phase image.

Table S3. *IM* (rad) of corrected SPR phase images overlapped with a circular mask

Added coma* / Correction modes	Hor.(cell1)	Vert.(cell1)	Hor.(cell2)	Vert.(cell2)
First six modes	0.51	0.69	0.54	0.72
Coma modes included	0.28	0.28	0.31	0.31

*Hor.: horizontal coma; Ver.: vertical coma. Coefficient set to 1.

References

1. Li, J. C. et al. Digital holographic reconstruction of large objects using a convolution approach and adjustable magnification. *Optics Letters* **34**, 572-574 (2009).
2. Kreis, T. M. Frequency analysis of digital holography with reconstruction by convolution. *Optical Engineering* **41**, 1829-1839 (2002).
3. Zhang, J. W. et al. Wavelength-multiplexing surface plasmon holographic microscopy. *Optics Express* **26**, 13549-13560 (2018).
4. Dai, S. Q. et al. Real-time and wide-field mapping of cell-substrate adhesion gap and its evolution via surface plasmon resonance holographic microscopy. *Biosensors and Bioelectronics* **174**, 112826 (2021).
5. Ren, Z. B., Zhao, J. L. & Lam, E. Y. Automatic compensation of phase aberrations in digital holographic microscopy based on sparse optimization. *APL Photonics* **4**, 110808 (2019).
6. Dai, S. Q. et al. Optical tweezers integrated surface plasmon resonance holographic microscopy for characterizing cell-substrate interactions under noninvasive optical force stimuli. *Biosensors and Bioelectronics* **206**, 114131 (2022).
